# Founder-specific inbreeding depression affects racing performance in Thoroughbred horses

**DOI:** 10.1038/s41598-018-24663-x

**Published:** 2018-04-18

**Authors:** Evelyn T. Todd, Simon Y. W. Ho, Peter C. Thomson, Rachel A. Ang, Brandon D. Velie, Natasha A. Hamilton

**Affiliations:** 1 School of Life and Environmental Sciences, The University of Sydney, Sydney, NSW 2006 Australia; 2 Department of Animal Breeding and Genetics, Swedish University of Agricultural Sciences, 75007 Uppsala, Sweden

## Abstract

The Thoroughbred horse has played an important role in both sporting and economic aspects of society since the establishment of the breed in the 1700s. The extensive pedigree and phenotypic information available for the Thoroughbred horse population provides a unique opportunity to examine the effects of 300 years of selective breeding on genetic load. By analysing the relationship between inbreeding and racing performance of 135,572 individuals, we found that selective breeding has not efficiently alleviated the Australian Thoroughbred population of its genetic load. However, we found evidence for purging in the population that might have improved racing performance over time. Over 80% of inbreeding in the contemporary population is accounted for by a small number of ancestors from the foundation of the breed. Inbreeding to these ancestors has variable effects on fitness, demonstrating that an understanding of the distribution of genetic load is important in improving the phenotypic value of a population in the future. Our findings hold value not only for Thoroughbred and other domestic breeds, but also for small and endangered populations where such comprehensive information is not available.

## Introduction

The Thoroughbred horse population is one of the largest closed populations of animals in the world. Thoroughbreds are extremely valuable because of the large amount of prizemoney on offer and the high residual value of superior athletes. All Thoroughbred horses trace their ancestry back to three paternal lines, due to the narrow bottleneck at the foundation of the population^1–3^. More than 300 years of breeding practices have produced signatures of selection in the 21^st^ century Thoroughbred population, contributing to the superior athleticism of the breed^4,5^. At the same time, these practices have increased levels of inbreeding and reduced the genetic diversity of Thoroughbreds compared with other domestic horse breeds^3,6,7^.

To our knowledge, there has been no detailed examination of the effects of inbreeding on the racing performance of Thoroughbred horses and the genetic load of the population. Genetic load, the presence of unfavourable genetic material, is a reflection of a population’s fitness because a higher genetic load leads to a lower mean fitness level^8^. A large proportion of genetic load consists of recessive deleterious mutations, known as mutational load. Inbreeding can expose mutational load because it increases an individual’s chance of inheriting two copies of recessive deleterious alleles from a common ancestor^8,9^. The subsequent decrease in fitness caused by these expressed recessive deleterious mutations is thought to be a major cause of inbreeding depression^10^. Other mechanisms believed to contribute to inbreeding depression include epistatic interactions and reductions in favourable heterozygosity^10,11^.

The inevitable effect of selection in a closed population is an increase in the level of inbreeding^12,13^. There is some evidence that continued inbreeding for selection can purge a population of some or all of its genetic load, such that new inbreeding events have negligible or even positive effects on phenotype^9^. Although some domestic and wild populations show signs of purging^14–16^, others still show strong signs of inbreeding depression even after multiple population bottlenecks and inbreeding events^17–19^. Purging is most likely to occur in populations under strong selection and slow rates of inbreeding, allowing deleterious alleles to be effectively eliminated rather than fixed by genetic drift^11,20^. Additionally, inbreeding for favourable phenotypic characteristics can have unexpected negative implications through deleterious alleles hitchhiking on regions of the genome under positive selection, thereby increasing their frequency in the population^21–23^.

Understanding the effects of selection is further complicated by the uneven distribution of genetic load in a population. Inbreeding to different ancestors can have varying effects on fitness, such that the total proportion of alleles identical by descent (IBD) might not be an accurate reflection of mutational load^24–26^. This raises the possibility that inbreeding in different pedigree lines has variable effects on genetic load in the Thoroughbred population.

The availability of extensive phenotypic and pedigree records, dating back to the late 18^th^ century, makes the Thoroughbred population ideal for studying the long-term, population-wide effects of selection on performance and genetic load. Here, we examine the effects of inbreeding on racing performance and mutational load in the Australian Thoroughbred population. Australia has the second-largest racing and breeding population in the world, containing approximately 15% of all Thoroughbreds^27^.

We analyse a sample of 135,572 individuals, representing all Thoroughbred horses that had one or more race starts in Australia between 2000 and 2011. A genealogy of these individuals, dating back to the founders of the population (*n* = 257,249), is also included in our analyses. Although some lines of pedigree are incomplete, we have comprehensive pedigree information for all individuals in the racing performance data set, making our inbreeding estimates highly accurate. The availability of extensive pedigree records not only allows us to study broad population trends over time, but also to determine whether the selection for optimal racing performance has alleviated mutational load. We use these data to measure inbreeding and ancestral coefficients for all individuals. We also identify the ancestors that have made the greatest genetic contributions, in order to understand better the distribution of mutational load in the population. For a representative subset of individuals, we perform high-density genotyping to determine whether inbreeding load is reflected at the genomic level.

## Results and Discussion

### The effects of inbreeding and purging on racing performance

**Figure 1:**
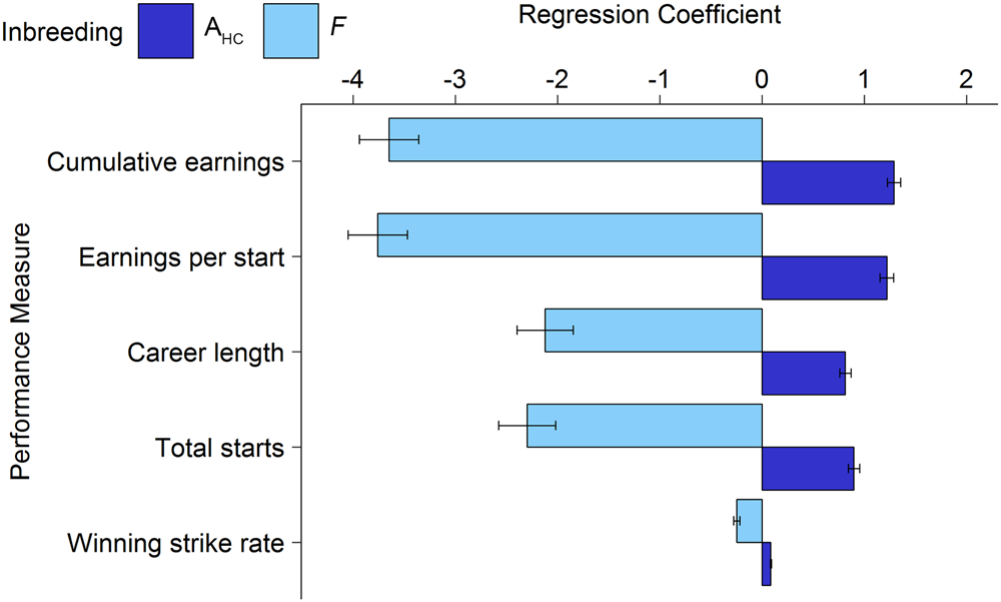
Regression coefficients showing the relationship between measures of racing performance and inbreeding in Thoroughbred horses (*n* = 135,572). All measures of racing performance have a negative relationship with *F* but a positive association with A_HC_. Error bars represent 1 standard error around the mean. Regression coefficients and standard errors were divided by the standard error of their respective traits. The relationship between each measure of inbreeding and racing performance was highly significant (P < 0.001).

Our analysis of data from 135,572 Thoroughbred horses revealed a strong negative relationship (all *P* < 0.001, Fig. 1) between Wright’s inbreeding coefficient, *F*, and five measures of racing performance that encompass a range of factors that contribute to exercise performance^28,29^. These included two measures that are based on the assumption that more successful individuals earn more prizemoney: cumulative prizemoney earnings and prizemoney earnings per start. We also included two measures of constitutional soundness: total number of race starts and career length. Finally, we accounted for consistency of performance with the measure winning strike rate.

The negative relationship between *F* and performance can be explained by a genetic load of partially deleterious alleles still being carried by the population. We expect that the alleles causing the observed inbreeding depression are more difficult to select out of the population than those with lethal or debilitating effects on juvenile or embryonic survival^10,21,30–32^. Population bottlenecks that occurred during the ancestry of the Thoroughbred, including the domestication of the horse^33^, and the foundation of the breed^2,3^, might have increased the frequency of deleterious alleles through genetic drift. It is also possible that continued inbreeding of the Thoroughbred population over the past 300 years has inadvertently increased the frequency of deleterious variants in the population, potentially through hitchhiking on selective sweep regions^13,21,23^. As a result of many generations of inbreeding, the average *F* of the 21^st^ century Thoroughbred population is 0.139 (*s* = 0.011).

In contrast with the results from Wright’s inbreeding coefficient, the ancestral history coefficient, A_HC_, showed a strong positive association with racing performance (all *P* < 0.001, Fig. 1). This statistic, described by Baumung, *et al*.^34^, counts the number of times that an allele has been IBD in an individual’s pedigree, thus providing a comprehensive reflection of selection for favourable traits over time. The A_HC_ statistic is based on the assumption that an allele that has been IBD multiple times in an individual’s pedigree is likely to have a neutral or positive effect on fitness. In contrast, an allele that is IBD for the first time is more likely to have a negative effect on fitness. Therefore, individuals with higher A_HC_ are more likely to contain larger proportions of alleles in their genomes that have been positively selected over many generations. It is possible for an individual with a comprehensive pedigree to have an A_HC_ greater than 1. As a consequence of the comprehensive and inbred pedigree, the reference population had average A_HC_ of 1.973 (*s* = 0.089).

The positive relationship between A_HC_ and all measures of racing performance is possibly due to the many generations of selective breeding that have increased the frequency of alleles associated with positive improvements with exercise physiology. These alleles will appear IBD more times in the pedigrees of each subsequent generation, thus driving up A_HC_ (Appendix S2). Our results indicate that inbreeding for selection has effectively increased the frequencies of favourable alleles, but has not completely eliminated genetic load from the population. Considering this finding, it is unsurprising that parts of the Thoroughbred genome show signatures of selective sweeps linked to genes related to athletic performance, including formation of muscular fibres, upregulation of mitochondrial activity, angiogenesis, brown adipose tissue formation, and lipid metabolism^5,35^. In agreement with our results, there is some evidence for selection improving racing performance in another horse breed, the Norwegian cold-blooded trotter^32^.

Both *F* and A_HC_ showed the strongest associations with cumulative earnings and earnings per start (Fig. 1). We expect that this is because these measures reflect not only talent, but also good constitution because horses that race more are more likely to win more prizemoney. The smallest regression coefficient was for winning strike rate, probably because this measure is a crude estimate of consistency and does not reflect the race class, or the finishing order of a horse on non-winning occasions.

### The estimated breeding values of the population over time

**Figure 2:**
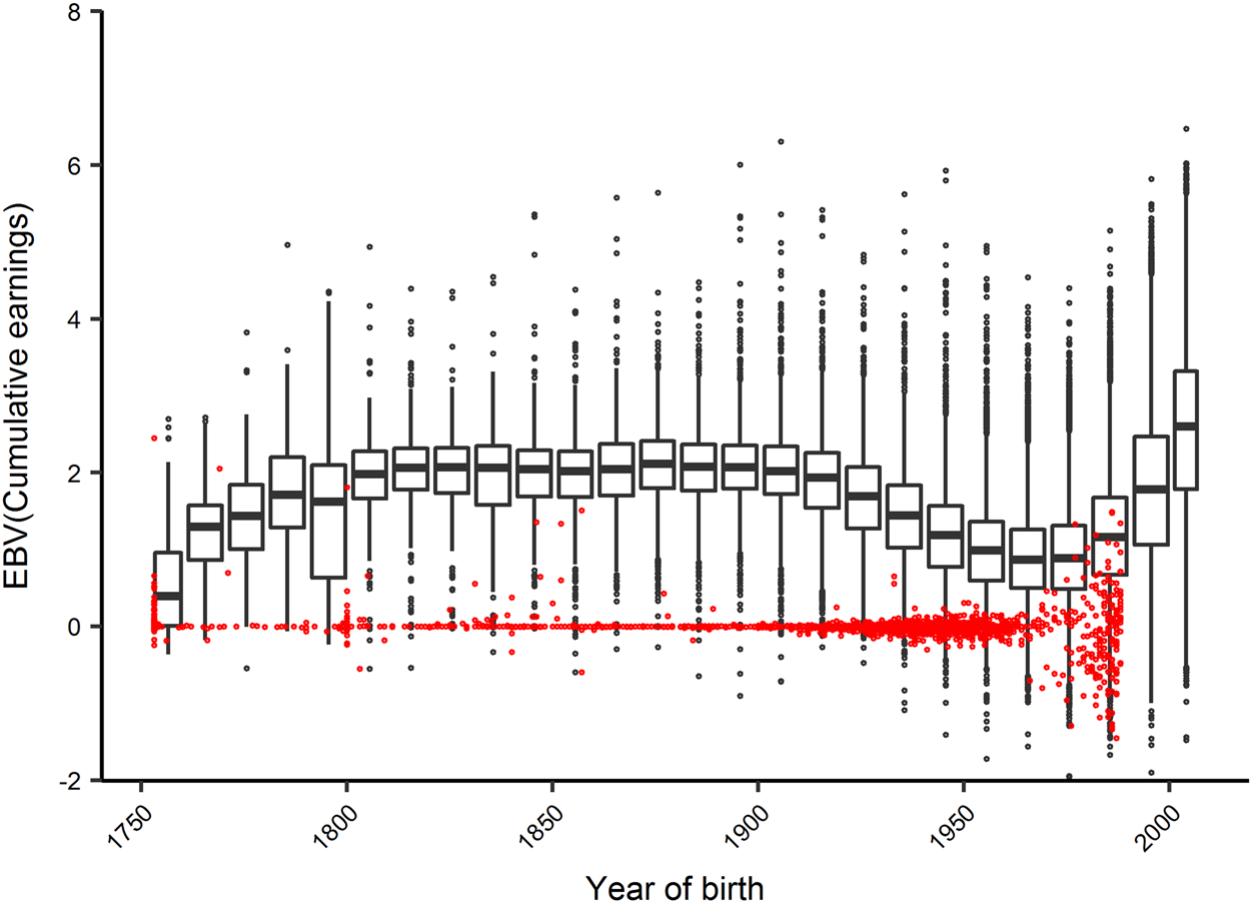
The distribution of estimated breeding values (EBVs) over time for Australian Thoroughbred horses (*n* = 257, 249), based on the cumulative earnings of 135,572 individuals that raced between 2000 and 2010. Bins were calculated over intervals of 0.2, with each bin representing a 10-year period. Individuals with unknown parents are shown in red. The EBV results for the other measures of racing performance follow the same trends and are included in the Appendix.

We found that selective breeding practices have not increased the overall performance levels of the population over time. We implemented a numerator relationship matrix in conjunction with a linear mixed model to account for additive genetic relationships between animals in the pedigree (Materials and Methods). Based on the racing performance of contemporary individuals (*n* = 135,572), we used this relationship matrix to calculate the estimated breeding values (EBVs) of all individuals in their pedigree (*n* = 257,249)^36,37^. The large increase in EBVs at the foundation of the population indicates that early selection events resulted in an initial jump in the frequency of favourable alleles (Fig. 2). After this initial increase, the distribution of EBVs remains constant; demonstrating that selective breeding from the early 19^th^ century was not effective in improving the racing performance of the population. The level of *F* has increased constantly during this time (Fig. S9), so we conclude that inbreeding has not effectively removed mutational load from the population. This explains why we observe strong inbreeding depression persisting in the contemporary population. We expect that this is due in part to a change in racing and training regimes over time that, in turn, has changed selection pressures on the population^38^. In the 18^th^ and early 19^th^ century, Thoroughbred races were held over a distance of several miles, with each horse participating in multiple heats on the same day. In the 20^th^ century, focus shifted to breeding sprinters and early developers for two-year-old racing^39^. Similarly, there was very little increase over time in the EBVs of Polish Warmblood horses despite selection for performance, indicating that intensive selection might be necessary to improve the mean value of complex quantitative traits in a population^40^.

The dip in EBVs between 1930 and 1980 can also be partly attributed to an increased number of individuals with unknown pedigree information, as shown in red on Fig. 2. This, together with the increased variability of EBVs during this period, could also be due to the presence of less successful pedigree lines that have not been purged from the modern population. We expect that the increase in the average EBV from 1980 onwards is partly due to the introduction of parental testing in the 1980s, leading to complete pedigrees for all registered individuals. The increasing trend in EBVs over recent generations indicates a possibility for future improvement in the population’s overall phenotypic quality.

### The uneven ancestral genetic contribution in the contemporary Thoroughbred population

Selective breeding practices are likely to result in uneven ancestral genetic contributions, favouring ancestors carrying beneficial alleles and leading to the extinction of less successful ancestral lines^25,41,42^. We found that a small number of ancestors in the early years of the breed formation accounted for much of the inbreeding coefficient in the modern Australian Thoroughbred population.

We found that 10 ancestors accounted for, on average, over 80% of the IBD alleles in the modern Australian Thoroughbreds (Table 1). Almost 20% of the IBD alleles in the contemporary population were attributed to a single individual, Herod. We selected these 10 ancestors because they provided the greatest marginal contributions to the individuals in our racing performance data (Appendix S4). The greatest marginal contributors are selected by first identifying the single ancestor with the greatest contribution to the population, and then subsequently finding the other ancestors that provide the greatest genetic contributions not accounted for by previously selected ancestors^43^ (Appendix S4). We then estimated the proportion of *F* (p*F*_*i*_) and A_HC_ (pA_HC*i*_) for each individual in our data set that is attributed to each of these ancestors^34,44^.

**Table 1:** The average partial *F* (p*F*_*i*_) and A_HC_ (pA_HC*i*_) coefficients of the contemporary population for the 10 ancestors with the greatest marginal contributions to the modern Australian Thoroughbred population (*n* = 135,572).

Ancestor name	Year of birth	Percentage contribution by each ancestor	
		*p*F	*p*A_HC_
Herod	1758	19.87	25.13
Eclipse	1764	11.5	12.97
St Simon	1881	8.74	4.58
Godolphin Arabian	1724	8.34	10.34
Touchstone	1831	7.73	5.79
Stockwell	1849	7.15	4.76
Rachel	1763	5.75	6.32
Snap	1750	5.41	5.77
Partner	1718	3.62	12.97
Roxana	1718	2.28	2.49
Total contribution		80.40	82.18

The final pair of columns shows the total average contribution of all 10 ancestors to the *F* and A_HC_ coefficients. All values are expressed as a percentage of the total *F* or A_HC_ value.

We identified these individuals as superior athletes that were also highly successful at stud. Historical records show that most of these individuals are closely related to each other (Fig. S8). One of them, Godolphin Barb, was one of the three foundation stallions of the breed in the early 18^th^ century^1^. He has been reported to contribute to 13.8% of the genetic makeup of British Thoroughbred horses^3^. Another of the foundation stallions, Eclipse, was identified as the source of a Y chromosome mutation that is near fixation in the modern Thoroughbred population^45^.

The 10 notable ancestors accounted for over 82% of the A_HC_ coefficient in their modern descendants (Table 1). We expected this relationship because these individuals appear many generations back in the pedigree of modern horses. For alleles inherited from them to have such a large contribution to *F*, they must appear IBD many times in the pedigrees of their descendants. In concordance with the principle of the A_HC_ coefficient, alleles that are found IBD multiple times in the pedigree are likely to have neutral or beneficial effects on fitness. These findings are reflected in the positive trends in *F* and A_HC_ over time in the population (Fig. S9).

### Uneven distribution of genetic load between different ancestors

**Figure 3:**
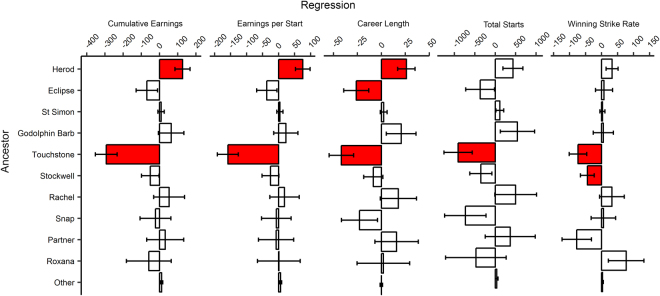
Inbreeding to different ancestors has variable effects on five measures of racing performance in modern Australian Thoroughbred horses. Partial inbreeding coefficients were calculated for the 10 ancestors with the greatest marginal contributions to the contemporary Australian Thoroughbred population. The relationship between each partial coefficient and inbreeding was analysed using regression coefficients from restricted maximum likelihood models. Error bars represent 1 standard error from the mean. This plot uses the same data set as in Fig. 1, but with each inbreeding coefficient split into partials. Red bars denote significant relationships.

We found evidence that founder-specific inbreeding depression differentially affects racing performance in the Australian Thoroughbred population (Fig. 3). We determined the distribution of genetic load between the 10 dominant ancestors by using linear mixed models to examine the relationship between partial inbreeding coefficients and racing performance. Genetic load may be unevenly distributed between different ancestors, such that inbreeding to different individuals can have a variable effect on fitness^24–26^. If inbreeding to a particular ancestor results in a reduction in the racing performance of their descendants, a higher proportion of the genetic load in the population can be attributed to them. The variation in genetic load between different ancestors indicates that inbreeding depression in Thoroughbreds is due to a small number of loci that have large effects on performance^24,25,46^.

We found that inbreeding resulting from four ancestors had significant effects on racing performance. Individuals with more IBD alleles attributed to Herod had greater cumulative earnings, earnings per start, and career length. This does not mean that increased inbreeding to Herod has had no negative effects on the phenotypic value of his descendants, but that overall they exhibit less inbreeding depression than other, equally inbred individuals^25^. Conversely, inbreeding to Eclipse, Stockwell, and Touchstone had negative effects on the racing performance of their descendants. We propose that these negative effects are partly due to the “cost of domestication”^23^, whereby inbreeding these individuals has inadvertently selected for deleterious alleles linked to sites that have undergone selective sweeps^21,22^.

Additionally, historical reports describe these stallions to be potential carriers of disease alleles, which may have predisposed their descendants to common conditions that reduce racing performance. Touchstone was reported by his contemporaries to have a number of conformational and behavioural issues^47^, which might also have contributed to the reduced level of performance in his descendants. Although Eclipse was a superior racehorse, his grandsire suffered from exercise-induced pulmonary haemorrhage (bleeding from the lungs)^48^. This hereditary condition reduces racing success^49,50^, and recurrent episodes result in a horse’s permanent ban from racing in Australia. Inheritance of this condition might be a contributing factor to the reduced career lengths of Eclipse’s descendants. Individuals with higher levels of inbreeding to Stockwell show reduced winning strike rates, although this might be a statistical abnormality because *P* = 0.04. However, Stockwell’s mother suffered from the congenital condition of laryngeal neuropathy (paralysis of the larynx)^51–53^, which may partly explain the observed reduction in performance.

We expect that most of these ancestors have passed on a mix of alleles with both positive and negative effects, such that inbreeding to them has the same effect as inbreeding to other individuals in the pedigree. However, it is also possible that two individuals inbred to the same ancestor could have inherited different sets of loci from different ancestral paths, making this ancestor’s effect on fitness variable between different descendants^46^. An instance of this has been found in cattle, where the occurrence of ectodermal dysplasia in a number of calves from unaffected parents was traced back to a *de novo* mutation in one bull^54^. The condition was only revealed through inbreeding of his descendants, when some of their progeny inherited two copies of the disease allele. This example demonstrates that inbreeding to a particular individual can have highly variable effects on fitness levels between their different descendants.

Considering the strong evidence for an uneven distribution in genetic load, we conclude that the majority of inbreeding depression is only due to small proportion of IBD alleles^25,42^. Consequently, we suggest that simply measuring the proportion of IBD alleles in the genome does not provide a comprehensive reflection of a population’s genetic load. Understanding the heterogeneous distribution of genetic load is important in assisting breeding decisions to minimize inbreeding to ancestors that negatively affect fitness^42^.

### Relationship between genome-based inbreeding coefficients and racing performance

In contrast with the pedigree-based estimates of inbreeding, we found that genomic measures of inbreeding showed no overall relationship with any measure of racing performance (Table 2). For a representative subset of the population (*n* = 122), we estimated genomic inbreeding levels as the proportion of the genome consisting of runs of homozygosity (*F*_ROH_). This method reflects inbreeding levels by capturing long, homologous tracts of DNA inherited from a common ancestor that have not been broken by recombination^55–59^. For our analyses, we selected minimal length thresholds of 5 Mb (*F*_ROH_5_) and 12 Mb (*F*_ROH_12_) to correspond to old and new inbreeding, respectively (Appendix S2).

**Table 2:** Regression coefficients of linear mixed estimating the association between five measures of racing performance and pedigree-based and genomic coefficients (*n* = 122).

	*F*_ROH_5_	*F*_ROH_12_	*F*	A_HC_
Cumulative earnings	1.95 (11.62)	5.05 (14.35)	−10.56 (19.74)	3.04 (3.51)
Earnings per start	0.63 (8.53)	2.30 (10.54)	−10.10 (14.55)	1.61 (2.58)
Career length	−0.69 (2.32)	−0.84 (2.86)	−3.26 (3.64)	0.54 (0.69)
Total starts	−10.61 (87.49)	−24.01 (107.94)	16.32 (142.13)	38.81 (25.81)
Winning strike rate	−2.17 (4.16)	−5.08 (5.14)	−17.37 (6.61)*	−0.43 (1.15)

Sex and year of birth were added as fixed effects and a numerator relationship matrix as a random effect in each model. Cumulative earning, earnings per start, and career length were log transformed for a normal distribution and analysed with a linear mixed model. Total starts was analysed using a Poisson generalized linear mixed model and winning strike rate using a binomial generalized linear mixed model. Inbreeding was measured using the pedigree measures of: Wright’s inbreeding coefficient (*F*) and the ancestral history coefficient (A_HC_). Genealogical inbreeding was measured as the proportion of runs of homozygosity (ROH) in the genome with the minimal lengths of 5MB (*F*_ROH_5_) and 12MB (*F*_ROH_12_). Standard errors are shown in parentheses. *P < 0.05; **P < 0.001.

For this smaller data set, however, we also found that the *F* and A_HC_ coefficients for these individuals also showed no relationship with performance (Table 2). Considering that this relationship was significant for a larger sample size, we conclude that a sample size of 122 was not sufficient to capture the relationship between inbreeding and performance. Our models were unable to account for a number of confounding environmental factors that could affect racing performance (such as training regime, jockey success, and foal-rearing process), so a large sample size is needed to tease out the underlying relationship between inbreeding and performance. There is also a large continuum between the best- and worst-performing individuals in such a large population that might not be captured by a small subset of individuals. Our findings indicate that caution should be exercised in studies of smaller populations.

Molecular estimates of inbreeding are often considered to be superior to genealogical measures because they account for the unpredictable nature of recombination and inaccurate pedigree-recording information. However, the parameters of *F*_ROH_ measurements should also be chosen carefully, so that they accurately reflect inbreeding levels. The accuracy of these estimations might be affected by inadequate SNP density^56,60^ and long tracts of ROH persisting in areas of low recombination^61,62^ (Appendix S2). Many studies use different parameters for genotyping densities, data trimming, and ROH, making comparisons between them difficult to draw.

We found that the correlation between *F*_ROH_ and *F* in our data set (Fig. S3) was lower than that reported in other domestic species^63,64^, which may partly explain the contrasting results. We found that a large proportion of the inbreeding coefficient in the Australian Thoroughbred population was accounted for by ancestors many generations back in the pedigree. Inbreeding to distant ancestors results in shorter ROH regions that might not be captured by the SNP density used in our analysis (Appendix S2).

For these reasons, we believe that for large populations with comprehensive pedigrees, genealogical measures of inbreeding can provide important inferences if the size of the pedigree is much larger than the number of individuals genotyped. The use of pedigree data allows inferences to be made for deceased individuals, for which genotyping might not be possible. Additionally, using a pedigree to analyse trends over time can be advantageous because it might not be possible to obtain molecular data for deceased individuals (such as the founders of the population). Pedigrees also provide the opportunity to estimate the effects of specific individuals over time on the fitness of their descendants.

## Conclusions

In this study, we have presented the effects of inbreeding and selection in a very large population with extensive phenotypic and pedigree records. Our analyses have shown that genetic load can still persist in a population even after many generations of inbreeding. However, we have also found evidence that multiple generations of inbreeding for selection can have positive effects on the overall genetic value of a population. We suggest that using EBVs whilst managing inbreeding levels will increase the efficiency of selection to reduce inbreeding depression in subsequent generations. Further, our findings highlight the need for caution in studies with small sample sizes because they can lead to inaccurate inferences about the effects of inbreeding.

We have also found evidence that the genetic load is unevenly distributed in the Thoroughbred population. This indicates that studies of inbreeding need to account for heterogeneity between different ancestors, because the total proportion of IBD alleles might not accurately reflect genetic load. Understanding the distribution of genetic load in the population will assist in breeding decisions to reduce disease alleles and improve the overall fitness of the population in future generations. Our findings open the possibility of evaluating the effects of particular individuals on the fitness of the population in order to improve phenotypic quality and reduce genetic load in the future.

## Materials and Methods

### Calculating pedigree-based inbreeding coefficients

Racing Australia provided race records for all individuals that had participated in a race start in Australia between 2000 and 2010 (*n* = 135,572). A genealogy of all horses born after 1970, dating back to the founders of the population, was provided by the Australian Stud Book (*n* = 500,477) (Appendix S1). We trimmed the pedigree file so that it only included the ancestors of the individuals in our data set, leaving a pedigree size of 257,249. We found that all individuals included in our analysis had a comprehensively recorded pedigree (an average of 24.60 discrete generational equivalents of known pedigree^65,66^). Before 1980, however, a small number of individuals appear in the stud book with no recorded pedigree^67,68^ (Appendix S6). These individuals accounted for 1.4% of the total ancestors included in our genealogy file, and mostly appear more than 6 generations back in the pedigree.

We estimated inbreeding levels for all individuals in the data set using Wright’s inbreeding coefficient (*F*)^69^. We used this traditional measure of quantifying inbreeding to allow our results to be compared with those from previous studies. We also used the pedigree data to estimate several ancestral inbreeding coefficients that account for genetic load (SI Materials and Methods, Appendix S1, S2)^18,34,70^. We selected the ancestral history coefficient (A_HC_) for further analysis because this measure counts the number of times that an allele has been IBD in an individual’s pedigree, thus providing a comprehensive reflection of selection for favourable traits over time^34^.

We calculated *F* and A_HC_ for all individuals in the pedigree using 10^6^ replications of simulated gene drops in GRain 1.0^34^ (Appendix S1). This method uses Mendelian segregation rules to simulate gene flow through a population by flagging each allele as it runs through the pedigree. These data are then used to estimate the probability-based inbreeding coefficients^71^. The accuracy of the results depends on the number of replications performed, which is proportional to the number of unlinked loci calculated in the analysis^34^. We checked the accuracy of our output by comparing *F* estimations using GRain with a deterministic approach as implemented by PEDIG^66^. Estimates from the two methods had a correlation coefficient of 0.99, indicating high accuracy of the inbreeding estimations by GRain.

We identified the 20 ancestors that provided the greatest marginal contributions to the population of 135,572 individuals by using iterations in the *prog_orig.f* program in PEDIG^43,66^. We then used GRain to calculate p*F* and pA_HC_ of each ancestor for each individual in our data set. Ten ancestors were chosen for further analysis, and their identities were determined using the Australian Stud Book and the online pedigree database (pedigreequery.com).

### Estimating inbreeding from genomic data

We selected a representative subset of individuals for high-density genotyping (*n* = 128). These individuals were selected to provide a reflection of different bloodlines in the population and a continuum of racing successes. We used these data to estimate the proportion of the genome consisting of runs of homozygosity (*F*_ROH_).

To estimate genome-based levels of inbreeding, we first extracted DNA from hair samples (collected under approval from University of Sydney Ethics Committee N00-2009-3-5109) using the Qiagen Gentra® Puregene® Tissue Kit (Qiagen, Redwood City, CA, USA). We genotyped 105 individuals using the Equine SNP70 BeadChip (Illumina, San Diego, CA, USA), which consists of 65,102 SNPs evenly distributed throughout the equine genome. Additionally, we typed 23 individuals on the Axiom Affymetrix SNP Chip (670,671 SNPs), when this higher density array became available at a later date. We used custom Perl scripts to extract only the SNPs that were common to these two panels, which we then used in further analyses.

SNP data were edited and analysed using PLINK 1.07^72^. The data were trimmed to be in concordance using the following parameters: minor allele frequency > 0.01; individual call rate > 0.9; and SNP call rate > 0.9^6,73^. This process yielded a final data set comprising 45,451 SNPs for each of 122 individuals. Additionally, we only analysed autosomal SNPs in order to exclude any bias between male and female.

We used these data to estimate the proportion of the genome consisting of runs of homozygosity (*F*_ROH_). To define the parameters of an ROH, we set the minimum density to 0.05 Mb/SNP and the largest gap to 1 Mb, in accordance with the settings used by Goddard, *et al*.^74^ and Silió, *et al*.^63^. We set the minimal number of SNPs in each ROH to 20, because our SNP coverage was approximately 1 SNP every 50 Mb, making this sufficient to distinguish an ROH of 1 Mb. ROH lengths were calculated as a proportion of total ROH length in relation to the total equine autosome size of 2,242,879,462 bp.

### Measuring racing performance

We selected five different measures of racing performance that account for talent, consistency, and constitutional soundness^28,29,75^. These measures were: cumulative earnings ($AU), earnings per start ($AU), career length (months), total number of race starts, and winning strike rate. Cumulative earnings and earnings per start favour talented individuals, because more prestigious races carry larger prizemoney purses. Career length and total starts favour individuals with good constitutions; individuals with health and conformational defects are unable to race for extended periods. Winning strike rate accounts for consistency in horses, because more talented horses are expected to win a higher proportion of their race starts (Appendix S3).

### Statistical analysis

The relationship between each measure of inbreeding and racing performance was analysed using (generalized) linear mixed models in ASReml-R 3.0^76^. We used the five measures of racing performance as outcome variables. Cumulative earnings, earnings per start and career length were analysed with linear mixed models. These variables were log-transformed; to accommodate zero-value in these measure, $100 was added to all career earnings and earnings per starts, and 1 month to all career length values. Total starts was analysed using a Poisson generalized linear mixed model and winning strike rate using a binomial generalized linear mixed model.

Each model included a predictor variable of either *F*, A_HC_, or *F* partitioned into partial coefficients for each of the 10 important ancestors, making a total of 15 models. Sex and year of birth were also included as predictor variables in each model. We also included a random animal effect that was associated with the numerator relationship matrix derived from the pedigree (n = 257, 249).

The significance of fixed effects was assessed using Wald tests. To allow comparisons of regression coefficients across different traits, the regression coefficients were divided by the standard deviation of their respective traits. EBVs were obtained from the fitted models. We summarised the EBV distributions over time in 10-year bins, which approximately represents one generation interval. We calculated the average generational interval to be 10.5 years using the *intgen.f* program from PEDIG^66^.

### Data Availability Statement

The data that support the findings of this study are available from Racing Australia and the Australian Stud Book. However, restrictions apply to the availability of these data, which were used under license for the current study, and so they are not publicly available. Data are, however, available from the authors upon reasonable request and with permission of Racing Australia. The data set can also be accessed from the public repositories of www.racingaustralia.horse and www.studbook.org.au.

## Electronic supplementary material

Supplementary Information

## References

[1] Weatherby, J. *An Introduction to a General Stud Book*. (Weatherby and Sons, 1791).

[2] Corbin LJ (2010). Linkage disequilibrium and historical effective population size in the Thoroughbred horse. Anim. Genet..

[3] Cunningham EP, Dooley JJ, Splan RK, Bradley DG (2001). Microsatellite diversity, pedigree relatedness and the contributions of founder lineages to Throughbred horses. Anim. Genet..

[4] Petersen JL (2013). Genome-Wide Analysis Reveals Selection for Important Traits in Domestic Horse Breeds. PLoS Genet..

[5] Gu J (2009). A genome scan for positive selection in Thoroughbred horses. PLoS ONE.

[6] Petersen JL (2013). Genetic Diversity in the Modern Horse Illustrated from Genome-Wide SNP Data. PLoS ONE.

[7] Metzger J (2015). Runs of homozygosity reveal signatures of positive selection for reproduction traits in breed and non-breed horses. BMC Genomics.

[8] Hedrick PW, Garcia-Dorado A (2016). Understanding InbreedingDepression, Purging, and Genetic Rescue. Trends in Ecol. Evol..

[9] Crnokrak P, Spencer CHB (2002). Perspective: purging the genetic load: a review of the experimental evidence. Evolution.

[10] Charlesworth D, Willis JH (2009). The genetics of inbreeding depression. Nat. Rev. Genet..

[11] Leroy G (2014). Inbreeding depression in livestock species: review and meta-analysis. Anim. Genet..

[12] Woolliams JA, Berg P, Dagnachew BS, Meuwissen THE (2015). Genetic contributions and their optimization. J. Anim. Breed. Genet..

[13] Zhang Q, Guldbrandtsen B, Bosse M, Lund MS, Sahana G (2015). Runs of homozygosity and distribution of functional variants in the cattle genome. BMC Genomics.

[14] Mc Parland S, Kearney F, Berry DP (2009). Purging of inbreeding depression within the Irish Holstein-Friesian population. Genet. Select. Evol..

[15] Moreno, E., Pérez-González, J., Carranza, J. & Moya-Laraño, J. Better fitness in the Capture Cuvier’s Gazelle despite inbreeding increase: evidence for purging? *PLoS ONE* **10** (2015). 10.1371/journal.pone.0145111 PMC4682998 26679703

[16] Laws RJ, Jamieson IG (2011). Is lack of evidence of inbreeding depression in a threatened New Zealand robin indicative of reduced genetic load?. Anim. Conserv..

[17] Kennedy ES, Grueber CE, Duncan RP, Jamieson IG (2014). Severe inbreeding depression and no evidence of purging in an extremely inbred wild species- the Chatham Island black robin. Evolution.

[18] Kalinowski ST, Hedrick PW, Miller PS (2000). Inbreeding depression in the Speke’s gazelle captive breeding program. Conserv. Biol..

[19] Boakes EH, Wang J, Amos W (2007). An investigation of inbreeding depression and purging in captive pedigreed populations. Heredity.

[20] Kristensen TN, Sørensen AC (2005). Inbreeding - lessons from animal breeding, evolutionary biology and conservation genetics. Anim. Sci..

[21] Marsden CD (2016). Bottlenecks and selective sweeps during domestication have increased deleterious genetic variation in dogs. Proc. Natl. Acad. Sci. USA.

[22] Cruz F (2008). The Legacy of domestication: accumulation of deleterious mutations in the dog genome. Mol. Biol. Evol..

[23] Lu J (2006). The accumulation of deleterious mutations in rice genomes: a hypothesis on the cost of domestication. Trends Genet..

[24] Lacy RC, Alaks G, Walsh A (1996). Hierarchical Analysis of Inbreeding Depression in Peromyscus polionotus. Evolution.

[25] Gulisija D, Gianola D, Weigel KA, Toro MA (2006). Between-founder heterogeneity in inbreeding depression for production in Jersey cows. Livest Sci.

[26] Casellas J, Varona L, Ibáñez-Escriche N, Quintanilla R, Noguera JL (2008). Skew distribution of founder-specific inbreeding depression effects on the longevity of Landrace sows. Genet Res (Camb).

[27] Australian Racing Board. 2015/2016 Australian Racing Fact Book. (Australian Racing Board, Sydney, Australia, 2016).

[28] Velie BD, Hamilton NA, Wade CM (2015). Heritability of racing performance in the Australian Thoroughbred racing population. Anim. Genet..

[29] Velie BD, Hamilton NA, Wade CM (2015). Performance selection for Thoroughbreds racing in Hong Kong. Equine Vet. J..

[30] Charlesworth B, Charlesworth D (1999). The genetic basis of inbreeding depression. Genet. Res..

[31] Hinrichs D (2007). Analysis of inbreeding depression in the first litter size of mice in a long-term selection experiment with respect to the age of the inbreeding. Heredity.

[32] Klemetsdal G (1998). The effect of inbreeding on racing performance in Norwegian cold- blooded trotters. Genet. Select. Evol..

[33] Schubert M (2014). Prehistoric genomes reveal the genetic foundation and cost of horse domestication. Proc. Natl. Acad. Sci. USA.

[34] Baumung R (2015). GRain: a computer program to calculate ancestral and partial inbreeding coefficients using a gene dropping approach. J. Anim. Breed. Genet..

[35] Hill EW, Gu J, McGivney BA, MacHugh DE (2010). Targets of selection in the Thoroughbred genome contain exercise-relevant gene SNPs associated with elite racecourse performance. Anim. Genet..

[36] Henderson CR (1975). Best linear unbiased estimation and prediction under a selection model. Biometrics.

[37] Bijma P, Woolliams JA (2000). Prediction of rates of inbreeding in populations selected on best linear unbiased prediction of breeding value. Genetics.

[38] Cassidy, R. Blood will tell In *The sport of kings: kinship, class and thoroughbred breeding in Newmarket* 140–157 (Cambridge University Press, 2002).

[39] Vamplew, W. Development of racing in *The turf: a social and economic history of horse racing* Ch. 1, 1–28 (Allen Lane, 1976).

[40] Borowska A, Wolc A, Szwaczkowski T (2011). Genetic variability of traits recorded during 100-day stationary performance test and inbreeding level in Polish warmblood stallions. Archiv fur Tierzucht.

[41] Dadar M, Mahyari SA, Rokouei M, Edriss MA (2014). Rates of inbreeding and genetic diversity in Iranian Holstein Cattle: Inbreeding, Genetic Diversity. Anim. Sci. J..

[42] Rodrigáñez J, Toro MA, Rodriguez MC, Silió L (1998). Effect of founder allele survival and inbreeding depression on litter size in a closed line of Large White pigs. Anim. Sci..

[43] Boichard D, Maignel L, Verrier É (1997). The value of using probabilities of gene origin to measure genetic variability in a population. Genet. Select. Evol..

[44] Nagy I (2010). Genetic diversity and population structure of the synthetic Pannon White rabbit revealed by pedigree analyses. J. Anim. Sci..

[45] Wallner B (2013). Identification of genetic variation on the horse Y chromosome and the tracing of male founder lineages in modern breeds. PLoS ONE.

[46] Casellas J, Piedrafita J, Caja G, Varona L (2009). Analysis of founder-specific inbreeding depression on birth weight in Ripollesa lambs. J. Anim. Sci..

[47] Fairfax-Blakeborough, J. *Northern turf history*, *vol. III* (J.A. Allen & Co, 1970).

[48] Erickson HH, Kindig CA, Poole DC (2000). Exercise-induced pulmonary hemorrhage: A new concept for prevention. J Equine Vet Sci..

[49] Morley PS, Bromberek JL, Saulez MN, Hinchcliff KW, Guthrie AJ (2015). Exercise‐induced pulmonary haemorrhage impairs racing performance in Thoroughbred racehorses. Equine Vet. J..

[50] Velie BD, Raadsma HW, Wade CM, Knight PK, Hamilton NA (2014). Heritability of epistaxis in the Australian Thoroughbred racehorse population. Vet. J..

[51] Ewart JC (1917). Horse-breeding and horse-racing. Nature.

[52] Boyko AR (2014). Genomic analysis establishes correlation between growth and laryngeal neuropathy in Thoroughbreds. BMC Genomics.

[53] Dupuis MC (2013). Detection of copy number variants in the horse genome and examination of their association with recurrent laryngeal neuropathy. Anim. Genet..

[54] Bourneuf E (2017). Rapid discovery of *de novo* deleterious mutations in cattle enhances the value of livestock as model species. Sci. Rep..

[55] Keller MC, Visscher PM, Goddard ME (2011). Quantification of inbreeding due to distant ancestors and its detection using dense single nucleotide polymorphism data. Genetics.

[56] Purfield DC, Berry DP, McParland S, Bradley DG (2012). Runs of homozygosity and population history in cattle. BMC Genet..

[57] Leutenegger A-L (2003). Estimation of the inbreeding coefficient through use of genomic data. Am. J. Hum. Genet..

[58] McQuillan R (2008). Runs of homozygosity in European populations. Am. J. Hum. Genet..

[59] Kristensen TN, Pedersen KS, Vermeulen CJ, Loeschcke V (2010). Research on inbreeding in the ‘omic’ era. Trends Ecol. Evol..

[60] Marras G (2015). Analysis of runs of homozygosity and their relationship with inbreeding in five cattle breeds farmed in Italy. Anim. Genet..

[61] Gibson J, Morton NE, Collins A (2006). Extended tracts of homozygosity in outbred human populations. Hum. Mol. Genet..

[62] Pemberton TJ (2012). Genomic patterns of homozygosity in worldwide human populations. Am. J. Hum. Genet..

[63] Silió L (2013). Measuring inbreeding and inbreeding depression on pig growth from pedigree or SNP-derived metrics. J. Anim. Breed. Genet..

[64] Kim E-S (2013). Effect of artificial selection on runs of homozygosity in U.S Holstein cattle. PloS ONE.

[65] Woolliams JA, Mäntysaari EA (1995). Genetic contributions of Finnish Ayrshire bulls over four generations. Anim. Sci..

[66] Boichard, D. In *7th World Congress on Genetics Applied to Livestock Production* Vol. paper 28–13 (Montpellier, France, 2002).

[67] O’Loghlen, F. *Champions of the Turf: By ‘Eurythmic’ (Frank O’Loghle**n)*. (F. H. Johnston Publishing Company, 1945).

[68] Ford, M. *The Australian Stud Book History*. Vol. 1 (The Australian Stud Book, 2006).

[69] Wright S (1922). Coefficients of inbreeding and relationship. Am. Nat..

[70] Ballou JD (1997). Ancestral inbreeding only minimally affects inbreeding depression in mammalian populations. J. Hered..

[71] Suwanlee S, Baumung R, Sölkner J, Curik I (2007). Evaluation of ancestral inbreeding coefficients: Ballou’s formula versus gene dropping. Conserv. Genet..

[72] Purcell S (2007). PLINK: A tool set for whole-genome association and population-based linkage analyses. Am. J. Hum. Genet..

[73] Ferencakovic M, Solkner J, Curik I (2013). Estimating autozygosity from high-throughput information: effects of SNP density and genotyping errors. Genet. Select. Evol..

[74] Goddard ME, Powell JE, Visscher PM (2010). Reconciling the analysis of IBD and IBS in complex trait studies. Nat. Rev. Genet..

[75] Velie BD, Wade CM, Hamilton NA (2013). Profiling the careers of Thoroughbred horses racing in Australia between 2000 and 2010. Equine Vet. J..

[76] Gilmour, A. R., Gogel, B. J., Cullis, B. R. & Thompson, R. *ASReml User Guide Release 3.0*. (VSN International Ltd, 2009).

