# Supplementary material for: Founder-specific inbreeding depression affects racing performance in Thoroughbred horses
Source: Sci Rep. 2018 Apr 18;8:6167. doi: 10.1038/s41598-018-24663-x (PMC5906619; doi:10.1038/s41598-018-24663-x)
Supplement: Supplementary file 1 — Supplementary Information [file 41598_2018_24663_MOESM1_ESM.pdf]

Supplementary Information

Founder-specific inbreeding depression affects racing performance in Thoroughbred horses.

Authors: Evelyn T. Todd[1*], Simon Y.W. Ho[1], Peter C. Thomson[1], Rachel A. Ang[1], Brandon D. Velie[2] and Natasha A. Hamilton[1].

[1] School of Life and Environmental Sciences, The University of Sydney, Sydney, NSW 2006, Australia

[2] Department of Animal Breeding and Genetics, Swedish University of Agricultural Sciences, 75007 Uppsala, Sweden.

Corresponding Author:

Evelyn Todd

School of Life and Environmental Sciences, The University of Sydney.

B19 - R.M.C. Gunn Building, Office 506
The University of Sydney
NSW 2006 Australia
T: +61421 541 218
E: evelyn.todd@sydney.edu.au

Table of Contents

**S1: Materials and Methods**	**3**
Calculating inbreeding coefficients	3
Calculating inbreeding from genomic data	3
**Results and Discussion**	**4**
S2: Comparison between different methods of measuring inbreeding and purging	4
Comparison between genealogical measures of inbreeding	4
Comparison between genomic measures of inbreeding	6
Comparison between genealogical and genomic inbreeding estimates	7
Which measure of inbreeding is best?	8
S3: Further output from linear mixed models	9
S4: The greatest ancestral contributions to the Thoroughbred population	11
S5: Whole-population inbreeding trends over time	13
S6: Pedigree structure and missing ancestors	14
**Additional References**	**15**
Fig. S1	19
Fig. S2	20
Fig. S3	21
Fig. S4	22
Fig. S5	23
Fig. S6	24
Fig. S7	25
Fig. S8	26
Fig. S9	27
Fig. S10	28
Fig. S11	29
Table S1	30

S1: Materials and Methods

Calculating inbreeding coefficients. Racing data for all horses who had participated in a race start between 2000 and 2010 was provided by Racing Australia (*n*=138,996). We pruned these data to exclude individuals who did not have their complete race records outlined in the data (e.g. individuals that might have just started their racing career in 2010), leaving a sample size of 135,572.

The genealogy of each individual born after 1970, dating back to the founders of the population, was also provided. The pedigree file consisted of 500,477 individuals and listed the sex, year of birth, and sire and dam identification for each individual. Pedigree loops were identified using *verif_ped* from PEDIG (1), and fixed by assigning the individual as a founder. F, F_{a_BAL}, F_{a_KAL} and A_{HC} coefficients were calculated by utilizing Grain 1.0 (2) to run 10^6 stochastic gene drops for each individual.

Calculating inbreeding from genomic data. We first estimated the proportion of homozygous SNPs in each individual's genome (F_H). We then calculated the proportion of each individual's genome made up of runs of homozygosity (F_{ROH}). We set the minimum number of SNPs in each run of homozygosity (ROH) to 20, because our SNP coverage was approximately 1 SNP every 50 Mb, making this sufficient to distinguish an ROH of 1 Mb. We used the minimal length parameters of 1, 2, 5, 8, 12 and 16 Mb, which correspond to approximately 50, 25, 10, 8, 5 and 3 generations, respectively (3). Pearson correlation coefficients using R were generated between all measures of inbreeding.

Results and Discussion

S2: Comparison between different methods of measuring inbreeding and purging

Comparison between genealogical measures of inbreeding. There are a number of ways to account for inbreeding and genetic load in a population. We compared a number of genealogical and genomic measures to determine the optimal coefficients to analyse inbreeding trends in individuals and populations. We used the stochastic gene-dropping program GRain to calculate genealogical inbreeding values (2) (See SI Materials and Methods). This simulation method allows accurate calculation of a number of ancestral inbreeding coefficients by accounting for alleles that are identical by descent (IBD) multiple times in the pedigree.

First, we calculated Wright's classical inbreeding coefficient (F) (4). Although F is the traditional method for calculating inbreeding in a population, it does not always reflect the genetic load of the population (5). There are a number of coefficients that can be calculated from pedigree data to account for purging of load and favourable selection. These measures rely on the principle that selection will remove deleterious alleles from the population, so alleles that are IBD multiple times in a pedigree are more likely to have neutral or positive effects on fitness.

Theoretically, a population can be purged of some or all of its genetic load, so that individuals with a high inbreeding coefficient may show little or no evidence of inbreeding depression (6-8). For this reason, accounting for potential purging provides a better reflection of genetic load than simply measuring F (9). A number of measures have been proposed, which all operate under the assumption that an allele which has been IBD more than once in an individual's pedigree is less likely to have a deleterious effect on phenotype than one that is IBD for the first time.

The first of these coefficients, proposed by Ballou (5), measures the proportion of the genome that has been IBD one or more times in previous generations:

$$F_{\text{a\_BAL}} = \frac{[F_{a(s)} + (1 - F_{a(s)})F_{(s)} + F_{a(d)} + (1 - F_{a(d)})F_{(d)}]}{2}$$

where $F_{a(s)}$ is the ancestral inbreeding coefficient of the sire, $F_{(s)}$ is the inbreeding coefficient of the sire, $F_{a(d)}$ is the ancestral inbreeding coefficient of the dam, and $F_{(d)}$ is the inbreeding coefficient of the dam. Importantly, this measure accounts for alleles that are IBD multiple times in each parent's pedigree, so $F_{\text{a\_BAL}}$ can be greater than 0 for individuals with F=0 (8).

In contrast, Kalinowoski's coefficient ($F_{\text{a\_KAL}}$) only accounts for alleles that are currently IBD, and have also been IBD in the past at least once (10). Hence, when F=0, $F_{\text{a\_KAL}}$ is also 0, resulting in a strong correlation between the two measures (Fig S1). A major shortcoming of $F_{\text{a\_BAL}}$ and $F_{\text{a\_KAL}}$ is that they only measure for alleles being IBD one or more times, so only account for high lethal, recessive alleles (11).

The ancestral history coefficient (A_{HC}) differs from $F_{\text{a\_BAL}}$ and $F_{\text{a\_KAL}}$ in that it accounts for the number of times an allele has been IBD in an individual's pedigree (2). This calculation is based on the assumption that the more times an allele has been IBD in an individual's pedigree, the more likely it is to have a neutral or beneficial effect on phenotype. It is therefore possible for an individual with a comprehensive and inbred pedigree to have an A_{HC}>1. A_{HC} is closely correlated with $F_{\text{a\_BAL}}$ because both measures account for all inbreeding events throughout the pedigree, although the former always holds a higher value because it quantifies the number of times an allele has been IBD (Fig. S1). This measure provides the most comprehensive and accurate reflection of purging in inbred individuals.

Comparison between genomic measures of inbreeding. The accuracy of genealogy-based inbreeding measures are highly reliant on the base population used. Each pedigree estimate assumes that the founders are unrelated, making them highly inaccurate for populations without reliable and comprehensive records. Our population has a complex pedigree with an average of 24.70 generations, allowing us to assume that these estimates are fairly accurate. However, the stochastic nature of recombination and the increase of allele frequencies through selection can lead to variability between probability-based and actual levels of autozygosity (approximately 2.43%) (12, 13). For this reason, there are increasing numbers of studies using high-density genomic information for inbreeding estimates (14). Genomic measures of inbreeding assume that increased levels of alleles IBD from common ancestors will result in higher levels of homozygosity (F_{H}) (14, 15).

To more accurately distinguish between alleles that are identical by state (IBS) and IBD, inbreeding levels are now often measured using runs of homozygosity (ROH). These long homozygous segments show evidence of a common ancestor, as they have not been broken down by recombination in meiosis (12, 16). The stochastic process of recombination means that shorter ROH segments correspond to ancient inbreeding, whereas larger ones correspond to recent consanguineous events (12, 17, 18). To differentiate between new and old inbreeding, we measured ROH with the minimal thresholds 1, 2, 5, 8, 12 and 16 Mb, which correspond to 50, 25, 10, 6, 5 and 3 generations, respectively (19). We also measured each individual's genomic level of homozygosity (F_{H}), because it does not distinguish between alleles that are IBS and IBD.

Pairwise relationships showed close correlations between each ROH threshold. There was very little difference between F_{ROH1} and F_{ROH2} (Fig. S2), possibly because the 45,451 SNP panel did

not provide adequate density to distinguish between these thresholds (3, 20, 21). There is increasing evidence that high-density panels are needed for accurate estimations of smaller ROH lengths (3, 20, 21). Some individuals in our dataset showed no evidence of recent inbreeding: six individuals did not have an ROH over 12 Mb (five generations), and 23 with none over 16 Mb (three generations).

Comparison between genealogical and genomic inbreeding estimates. As we expected, there are some correlations between pedigree- and genomic-based measures. *F* showed the closest correlation with F_{ROH16} (0.35, Fig. S3), probably reflecting recent inbreeding events (12, 22). Shorter ROH regions may not always correspond to autozygous segments, or may not be detected due to insufficient SNP coverage, explaining their lower correlations with *F* (21). Interestingly, our studies show a lower correlation between *F* and F_{ROH} than many other studies, with reported correlations of ~0.7 (22, 23). This is probably because much of *F* in the Thoroughbred population is attributed to individuals many generations back. The SNP density used in our analysis was probably not comprehensive enough to capture these distant inbreeding events.

The close relationship between $F_{\mathrm{a\_Kal}}$ and *F* measures has resulted in similar correlations with ROH coefficients (Fig. S3). Since A_{HC} and $F_{\mathrm{a\_Bal}}$ reflect levels of purging, rather than the proportion of the genome that is IBD, it is unsurprising they show no significant relationship with any genomic inbreeding measures. F_{H} had no relationship with any pedigree-based measure of inbreeding, indicating that its failure to distinguish between IBD and IBS alleles makes it a crude measure of inbreeding (Fig. S3).

Which measure of inbreeding is best? One major shortcoming that we have identified in using ROH estimations to quantify inbreeding is the lack of concordance in the parameters used to define an ROH (24), making it difficult to compare results between studies. For example, some studies allow for one heterozygous SNP in an ROH because of genotyping errors, whereas others believe that it makes estimations inaccurate, particularly if the heterozygous SNP is at the end of the ROH (24). Additionally, a 50k SNP panel may not be sufficiently dense to accurately detect ROH, and increasing panel density can identify different ROH regions (3, 20, 21). Additionally, the discovery of long ROH segments in outbred human populations (25) brings into question their accuracy for capturing levels of inbreeding. Recombination 'hotspots' and high linkage disequilibrium are proposed to account for these long IBD segments persisting for many generations (21, 25, 26). Considering that the Thoroughbred population has one of the highest linkage disequilibrium rates of any domestic animal population (27), this makes the accuracy of using ROH measurements to reflect inbreeding levels in the population questionable.

In contrast, F is a well-known and widely used method, so comparisons between different studies are much easier to make (although the accuracy and number of generations of the pedigree can confound these results). For populations with deep and complex pedigrees, such as the Thoroughbred population, F allows us to estimate whole-population inbreeding levels, as well as those for deceased individuals. In this respect, F is highly useful for studying inbreeding trends over time and predicting future implications for the population. However, F does not account for factors such as genetic drift and selection in the population, making ROH advantageous in this respect (17). For this reason, using F_{ROH} measures may be the most accurate way of measuring individual inbreeding levels in the Thoroughbred population.

Additionally, pedigree information can be used to estimate levels of purging and account for the selection of favourable alleles in a population. However, F_{a_Bal} and F_{a_Kal} do not effectively detect the purging of mildly deleterious alleles (11, 28). Therefore, we propose that the best coefficient for quantifying purging is A_{HC}. In a deep and complex pedigree (such as the Thoroughbred pedigree), this coefficient best captures the effectiveness of selective breeding practices in increasing the frequency of favourable alleles, and the purging of highly and mildly deleterious alleles.

Consequently, we have chosen F and A_{HC} measures for the further analysis of the effects of inbreeding on fitness. We have also chosen ROH thresholds of 5 and 12 Mb (corresponding to 10 and 5 generations, respectively). We chose the 5 Mb threshold to account for old inbreeding: with the SNP panel used in our study, any ROH estimate below 5 Mb may not truly reflect ROH coverage. We have also chosen the 12 Mb threshold to reflect new inbreeding, as 10% of the individuals in our dataset did not have any SNPs in the 16 Mb category.

S3: Further output from linear mixed models.

In our analyses we implemented multiple measures of racing performance to account for talent, consistency, and constitutional soundness. The first measure, cumulative earnings, accounts for the amount of prizemoney a Thoroughbred earns throughout their racing career, and is based on the assumption that an individual's ability will be reflected by the amount of prizemoney that they earn. However, this measure can favour individuals that perform inconsistently, but win one big prizemoney race in their career. For this reason, we included earnings per start, which favours individuals that perform consistently well in high-class races. Individuals that only

contest in one or two race starts in their lifetime could still have high cumulative earnings and earnings per start, so we also included career length as a performance measure. Career length does not account for long breaks between race starts on account of injuries or poor recovery, so total number of starts was also included in the analysis. Lastly a measure of a horse's consistency was also included as winning strike rate, because top-class individuals should win most, if not all, of their races. Consequently, these measures are all correlated (S4).

We found that sex and year of birth significantly affected all measures of racing performance. In all models, female horses had lower levels of performance. For prizemoney measures, this discrepancy is probably due to bigger, stronger males being able to win more races with higher prizemoney. This probably also accounts for males having a higher winning strike rate, as females racing against males will have less chance of winning. The lower total starts and career length observed for female horses can be explained by female horses being retired earlier for breeding purposes. Although some stallion prospects may also be retired early to stud, castrated males will continue racing for longer due to having no residual breeding value.

We used the linear mixed models to estimate the predicted values of each racing performance measure over a range of F and A_{HC} coefficients (Fig S5). These predictions follow the trends seen in the regression coefficients, with increasing F decreasing performance, and increasing A_{HC} enhancing it.

Using the numerator relationship matrix incorporated into the linear mixed models, we calculated the estimated breeding values (EBVs) for all individuals in the pedigree. The extensive pedigree information available for each individual in our sample increases the accuracy of our EBV information. However, it has been reported that phenotypic information over multiple generations will also increase the accuracy of EBV estimates (29).

We found that the distribution of EBVs in the population has changed over time (Fig. S6). The EBVs for each phenotypic measure showed similar trends, probably because of the correlations between measures. For this reason, we only present EBVs for cumulative earnings in the main body of the paper.

S4: The greatest ancestral contributions to the Thoroughbred population.

We determined the 20 ancestors with the greatest marginal contributions to the current population using iterations as implemented by PEDIG 1.0 (30). Calculating ancestral contributions rather than only contributions from founders accounts for bottlenecks in the pedigree, and is particularly advantageous in our population because it allows us to estimate and understand the contributions of particularly successful and popular breeders to the current Thoroughbred population.

Marginal contributions account for relationships between ancestors by finding the greatest contributor to the population, then finding the contributions of other individuals not accounted for by the already selected individuals (1). Founders and individuals near the top of the pedigree will be favoured in marginal contribution estimations, as individuals further down in the pedigree will have their contributions diluted by redundancy if their ancestors have also made large genetic contributions to the population, leading to a large difference between their raw and marginal contributions.

In this respect, marginal contributions are advantageous when modelling the heterogeneity of inbreeding depression. However, it is important to note that an ancestor will pass on different sets of genes to each of its descendants (31), so marginal contributions could overestimate

redundancies in the pedigree, as close relatives may have inherited completely different genetic information from the same common ancestor. We found large differences in the raw and marginal contributions of some ancestors in our dataset (Fig. S7, Table S1), such that selecting individuals based on raw contributions would result in a largely different sample space.

All of the greatest marginal contributors in our dataset are closely related, some of them sharing common ancestors, and others that were mated to produce a number of successful and influential progeny (Figure S8). The large contributions made by these close relatives demonstrate the narrow population bottleneck from which the breed has originated, mirrored in the initial large increase in the *F* coefficient at the foundation of the breed (Figure S9).

We found that many of these individuals were reported to be highly influential sires and dams in the early days of the Thoroughbred breed formation. Historically, there are considered to be four great sire lines responsible for the early formation of the Thoroughbred breed. Two of these, Herod and Eclipse, feature as the first and third greatest marginal contributors. The third, Highflyer is the son of Herod and Rachel, and the fourth, Matchem, is the grandson of Godolphin Arabian, Roxana and Brown Farewell. Highflyer's remaining grandparent Croft's Partner was not featured in the top ten contributors because his contribution has been diluted by the inclusion of his son, Partner.

Of the 20 ancestors analysed for their partial contributions, five contributed to over 5% of the genomes of the current population and ten contributed to over 2% (Figure S1). We selected the top ten individuals for further analysis because they all had a p*F*i of over 0.005.

S5: Whole-population inbreeding trends over time.

As expected in a closed population with a small number of dominant founders (32), *F* has increased consistently since the foundation of the Thoroughbred population (Figure S9a). A_{HC} values have also increased in the population over time (Figure S9b). The exponential increase of A_{HC} over time agrees with our previous findings that selection for alleles contributing favourably to performance has increased their frequency over time. This result is in concordance with the positive relationship found between A_{HC} and performance, indicating that an individual with a higher A_{HC} has a greater accumulation of these favourable alleles in their genome.

Of the 135,572 individuals included in our racing performance analysis, the average *F* was 0.139 and the average A_{HC} was 1.973. The large difference in these values further demonstrates that the selection for favourable alleles derived from the individuals in the early breed formation has increased their frequency over time.

We found that *F* levels rapidly increased after the bottleneck at the foundation of the breed. *F* then increased slowly in relation to A_{HC} over later generations (Figure S9c). There is a collection of individuals from 1930 onwards that have a lower *F* level than the majority of the population. These are the result of a parent that has an unknown pedigree or from an outbreeding event, often with one parent originating from a different continent. Although the *F* of these individuals is low, or 0, most of them have an A_{HC} value above 0. This indicates that both parents have inbreeding events in their own pedigree, which is not captured in *F* (see S2). We suggest that analysing both *F* and A_{HC} is needed to thoroughly examine inbreeding trends and effects in a population over time.

S6: Pedigree structure and missing ancestors

Although the individuals in our analysis trace back most of their pedigree lines to the founders of the population, a small number of ancestors have incomplete pedigree information. There are a number of reasons why their pedigrees are not completely recorded [33]. Firstly, an ancestor may not be registered in the stud book because its owner could not pay the stud book fees. This was particularly relevant during the Great Depression in the 1930s. Before 1980, it was acceptable in Australia for horses to be registered for racing without having a complete pedigree in the stud book. This was based on the assumption that to be competitive in Thoroughbred races, these horses would have to be of Thoroughbred origin. Pedigree records of some horses were lost when they were shipped from England to Australia in the early 19th century. For horses with American bloodlines, many pedigree records were lost during the American Civil War. Additionally, when DNA testing was introduced in the late 20th century, one individual was found to have false parentage. The proportion of ancestors with missing pedigree information by year is displayed in Fig. S10. These individuals accounted for 1.4% of the total ancestors in our pedigree file.

We estimated the proportion of missing ancestors by generation for all individuals used in our racing performance analysis (Fig. S11). No individuals in the racing performance data set have missing parents, and most individuals (80%) have a complete ancestry up to 6 generations (Fig. S11). Considering that the majority of F is captured in the first 6 generations of a pedigree[22], we consider that this pedigree structure makes the inbreeding estimates used in our analyses highly accurate.

Additional References

1. Boichard D, Maignel L, & Verrier É (1997) The value of using probabilities of gene origin to measure genetic variability in a population. *Genet Select Evol* 29(1):5-23.
2. Baumung R, et al. (2015) GRain: a computer program to calculate ancestral and partial inbreeding coefficients using a gene dropping approach. *J Anim Breed Genet* 132(2):100-108.
3. Ferencakovic M, Solkner J, & Curik I (2013) Estimating autozygosity from high-throughput information: effects of SNP density and genotyping errors. *Genet Select Evol* 45(1):42-42.
4. Wright S (1922) Coefficients of inbreeding and relationship. *Am Nat* 56(645):330-338.
5. Ballou JD (1997) Ancestral inbreeding only minimally affects inbreeding depression in mammalian populations. *J Hered* 88(3):169-178.
6. Laws RJ & Jamieson IG (2011) Is lack of evidence of inbreeding depression in a threatened New Zealand robin indicative of reduced genetic load? *Anim Conserv* 14(1):47-55.
7. Moreno E, Pérez-González J, Carranza J, & Moya-Laraño J (2015) Better fitness in the Capture Cuvier's Gazelle despite inbreeding increase: evidence for purging? *PLoS One* 10(12).
8. Mc Parland S, Kearney F, & Berry DP (2009) Purging of inbreeding depression within the Irish Holstein-Friesian population. *Genet Select Evol* 41(1):16.
9. Woolliams JA, Berg P, Dagnachew BS, & Meuwissen THE (2015) Genetic contributions and their optimization. *J Anim Breed Genet* 132(2):89-99.

10. Kalinowski ST, Hedrick PW, & Miller PS (2000) Inbreeding depression in the Speke's gazelle captive breeding program. *Conserv Biol* 14(5):1375-1384.

11. Boakes E & Wang J (2005) A simulation study on detecting purging of inbreeding depression in captive populations. *Genet Res* 86(2):139-148.

12. Keller MC, Visscher PM, & Goddard ME (2011) Quantification of inbreeding due to distant ancestors and its detection using dense single nucleotide polymorphism data. *Genetics* 189(1):237-249.

13. Carothers AD, et al. (2006) Estimating human inbreeding coefficients: comparison of genealogical and marker heterozygosity approaches. *Ann Hum Genet* 70(5):666-676.

14. Kristensen TN, Pedersen KS, Vermeulen CJ, & Loeschcke V (2010) Research on inbreeding in the 'omic' era. *Trends Ecol Evol* 25(1):44-52.

15. Leutenegger A-L, et al. (2003) Estimation of the inbreeding coefficient through use of genomic data. *Am J Hum Genet* 73(3):516-523.

16. McQuillan R, et al. (2008) Runs of homozygosity in European populations. *Am J Hum Genet* 83(3):359-372.

17. Curik I, Ferencakovic M, & Solkner J (2014) Inbreeding and runs of homozygosity: a possible solution to an old problem. *Livestock Science* 166(1):26-34.

18. Lencz T, et al. (2007) Runs of homozygosity reveal highly penetrant recessive loci in schizophrenia. *Proc Natl Acad Sci U S A* 104(50):19942-19947.

19. Ferenčaković M, et al. (2013) Estimates of autozygosity derived from runs of homozygosity: empirical evidence from selected cattle populations. *J Anim Breed Genet* 130(4):286-293.

20. Purfield DC, Berry DP, McParland S, & Bradley DG (2012) Runs of homozygosity and population history in cattle. *BMC Genet* 13:70.

21. Marras G, et al. (2015) Analysis of runs of homozygosity and their relationship with inbreeding in five cattle breeds farmed in Italy. *Anim Genet* 46(2):110-121.

22. Silió L, et al. (2013) Measuring inbreeding and inbreeding depression on pig growth from pedigree or SNP-derived metrics. *J Anim Breed Genet* 130(5):349-360.

23. Kim E-S, et al. (2013) Effect of artificial selection on runs of homozygosity in U.S Holstein cattle. *PloS One* 8(11):e80813.

24. Gurgul A, et al. (2014) The application of genome-wide SNP genotyping methods in studies on livestock genomes. *J Appl Genet* 55(2):197-208.

25. Gibson J, Morton NE, & Collins A (2006) Extended tracts of homozygosity in outbred human populations. *Hum Mol Genet* 15(5):789-795.

26. Pemberton TJ, et al. (2012) Genomic patterns of homozygosity in worldwide human populations. *Am J Hum Genet* 91(2):275-292.

27. Corbin LJ, et al. (2010) Linkage disequilibrium and historical effective population size in the Thoroughbred horse. *Anim Genet* 41(2):8-15.

28. Boakes EH, Wang J, & Amos W (2007) An investigation of inbreeding depression and purging in captive pedigreed populations. *Heredity* 98(3):172-182.

29. Yang H & Su G (2016) Impact of phenotypic information of previous generations and depth of pedigree on estimates of genetic parameters and breeding values. *Livestock Science* 187:61-67.

30. Boichard D (2002) Pedig: a fortran package for pedigree analysis suited to large populations. in *7th World Congress on Genetics Applied to Livestock Production* (Montpellier, France).

31. Casellas J, Piedrafita J, Caja G, & Varona L (2009) Analysis of founder-specific inbreeding depression on birth weight in Ripollesa lambs. *J Anim Sci* 87(1):72-79.

32. Hedrick PW, Hoeck PEA, Fleischer RC, Farabaugh S, & Masuda BM (2016) The influence of captive breeding management on founder representation and inbreeding in the ʻAlalā, the Hawaiian crow. *Conserv Genet* 17(2):369-378.

33. Ford, M. *The Australian Stud Book History*. Vol. 1 (The Australian Stud Book, 2006).

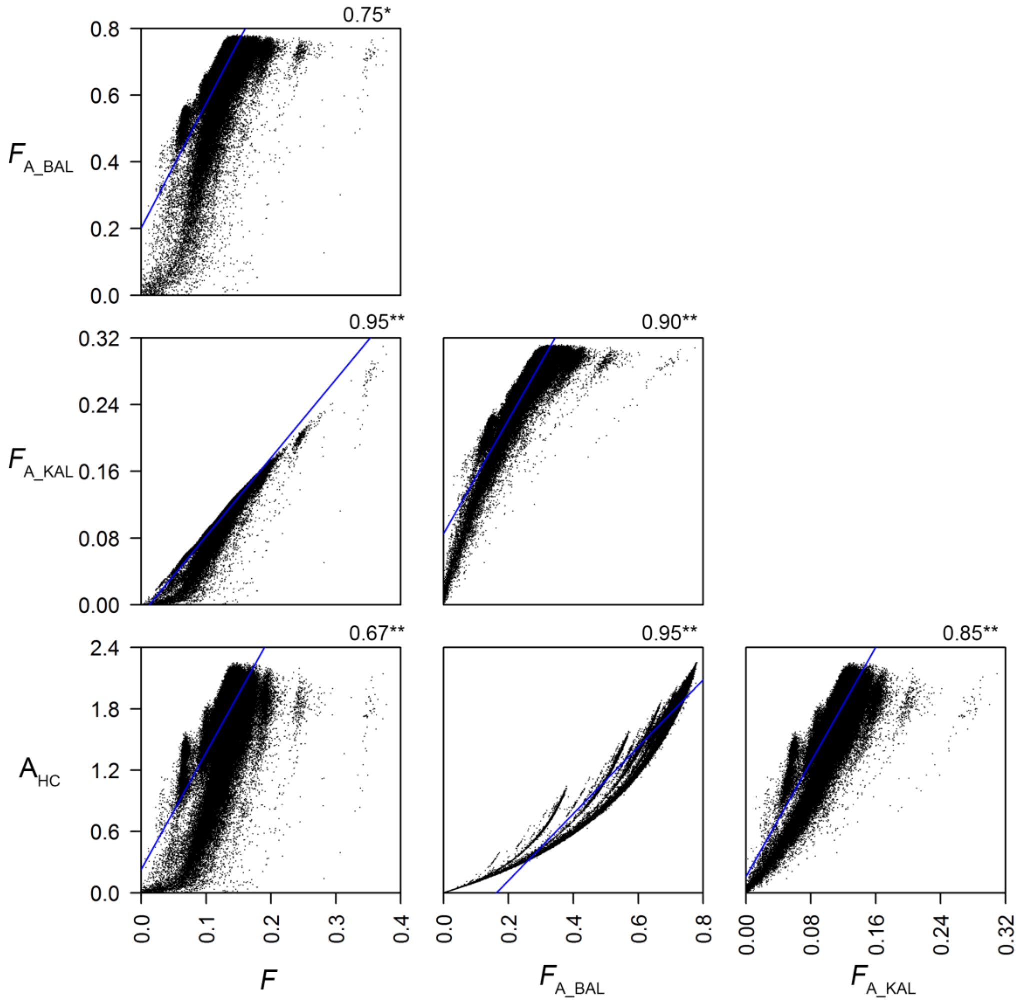

Fig. S1: Correlations between pedigree-based inbreeding measures estimated for Australian Thoroughbred racehorses (*n*=500,477). The inbreeding measures compared were: F, F_{A_BAL}, F_{A_KAL} and A_{HC}. Linear regressions are represented by blue lines. Pearson correlation coefficients are presented at the top-right of each graph. P-values <0.05 are marked with *, and P-values <0.001 with **.

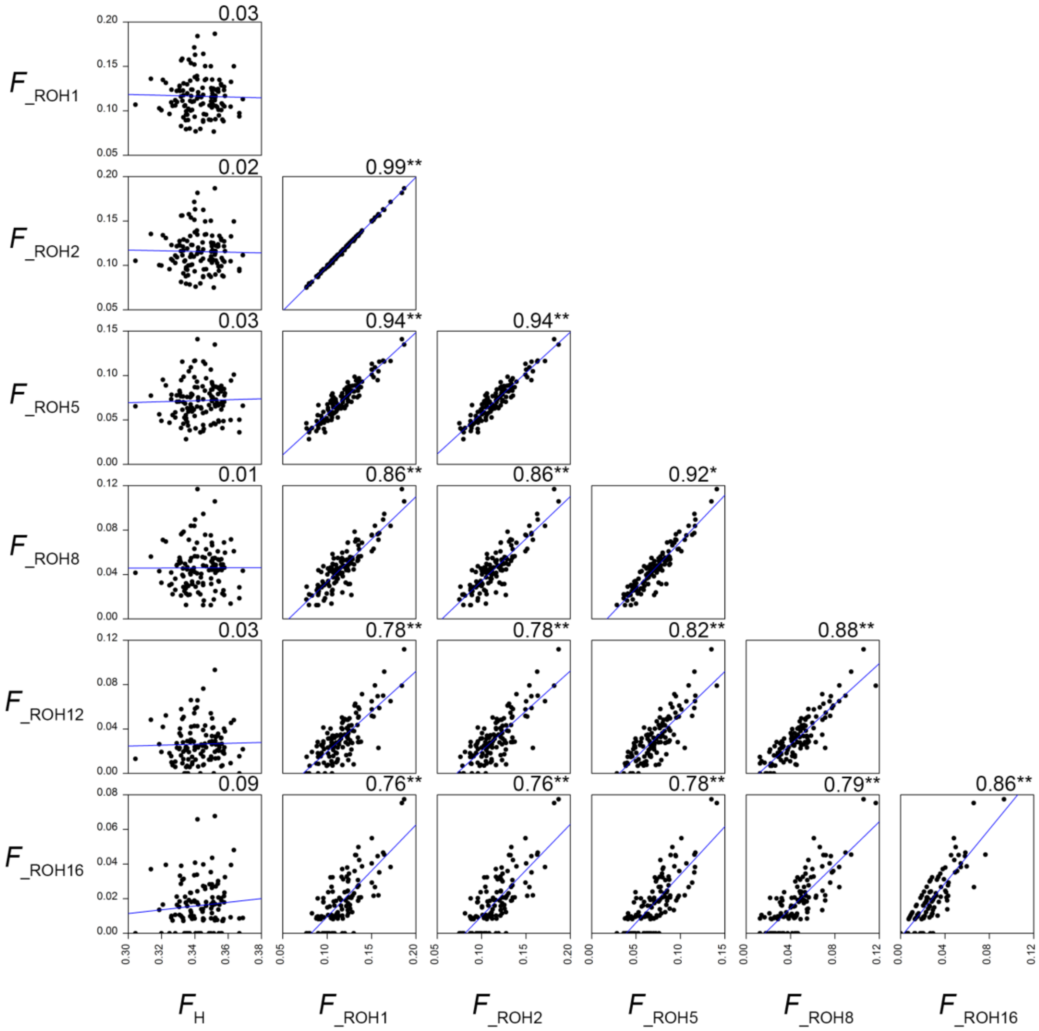

Fig. S2: Correlations between SNP-based inbreeding measures estimated for a representative subset of the modern Australian Thoroughbred population (*n*=122). The genomic measures of inbreeding levels compared were: F_{H}, $F_{\_ROH1}$, $F_{\_ROH2}$, $F_{\_ROH5}$, $F_{\_ROH8}$, $F_{\_ROH12}$, and $F_{\_ROH16}$. Linear regressions are represented by blue lines. Pearson correlation coefficients are presented in the top-right of each graph. P-values <0.05 are marked with *.

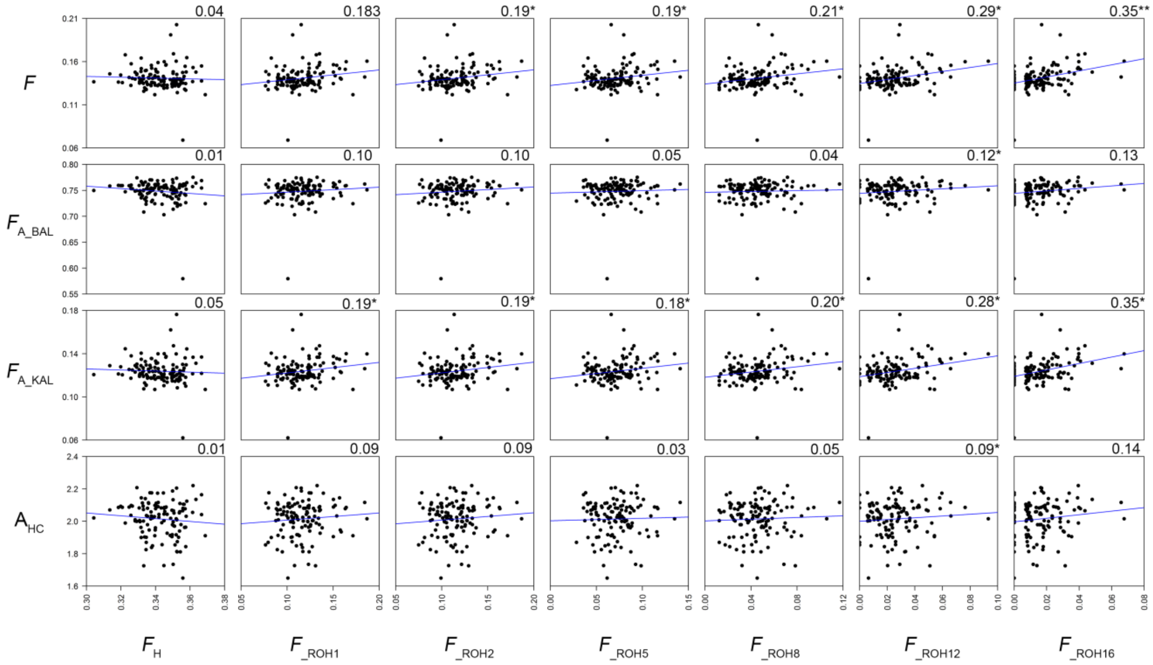

Fig. S3: Correlations between SNP- and pedigree-based inbreeding measures for a representative subset of the modern Australian Thoroughbred population (*n*=122). The pedigree-based inbreeding measures compared were: F, F_{A_BAL}, F_{A_KAL} and A_{HC}. The genomic measures of inbreeding levels compared were: F_H, $F_{\_ROH1}$, $F_{\_ROH2}$, $F_{\_ROH5}$, $F_{\_ROH8}$, $F_{\_ROH12}$, and $F_{\_ROH16}$. Linear regressions are represented by blue lines. Pearson correlation coefficients are presented in the top-right of each graph. P-values <0.05 are marked with *, and P-values <0.001 with **.

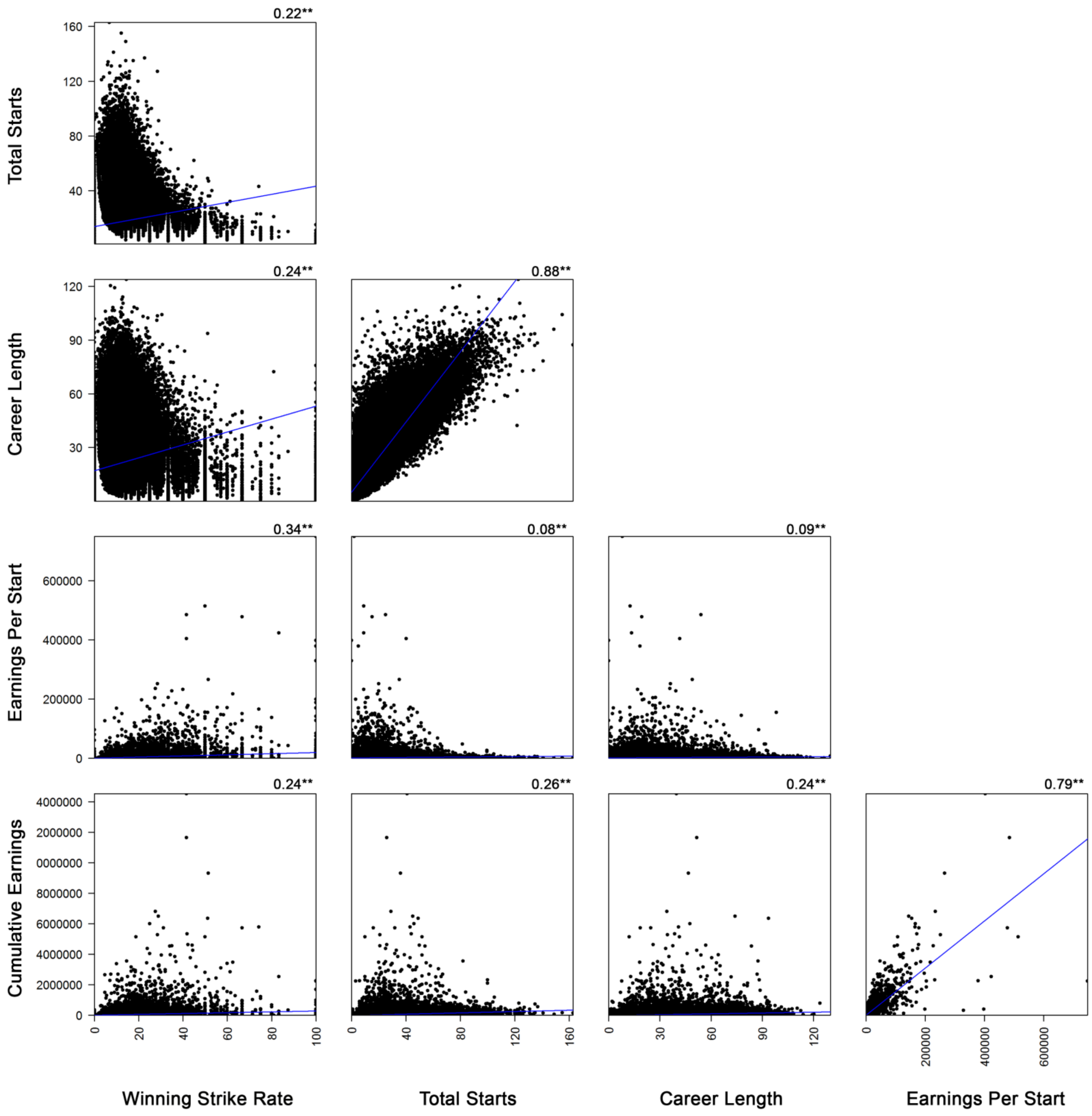

Fig. S4: Correlations between the five measures of racing performance used in the linear mixed model analysis (n=135,572). The measured analysed are: cumulative earnings ($AU), earnings per start ($AU), career length (in months), total number of starts and winning strike rate. Linear regressions are represented by the blue lines. Pearson correlation coefficients are presented in the top-right of each graph. P-values <0.001 are marked with **.

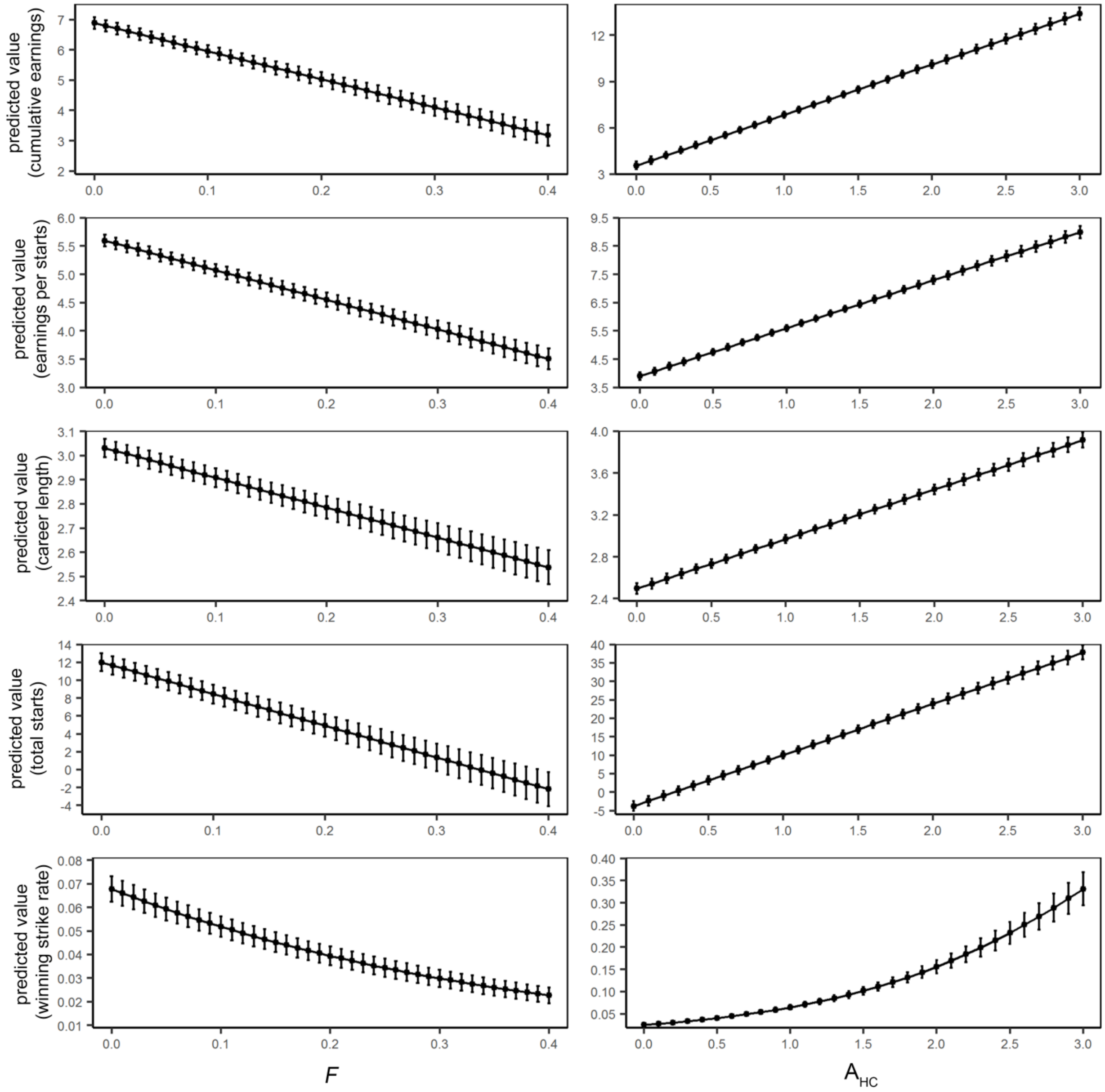

Fig. S5: Predicted values of the effects of *F* and A_{HC} on performance (*n*=135, 572). Estimates derived from linear mixed models. Error bars represent 1 standard error from the mean.

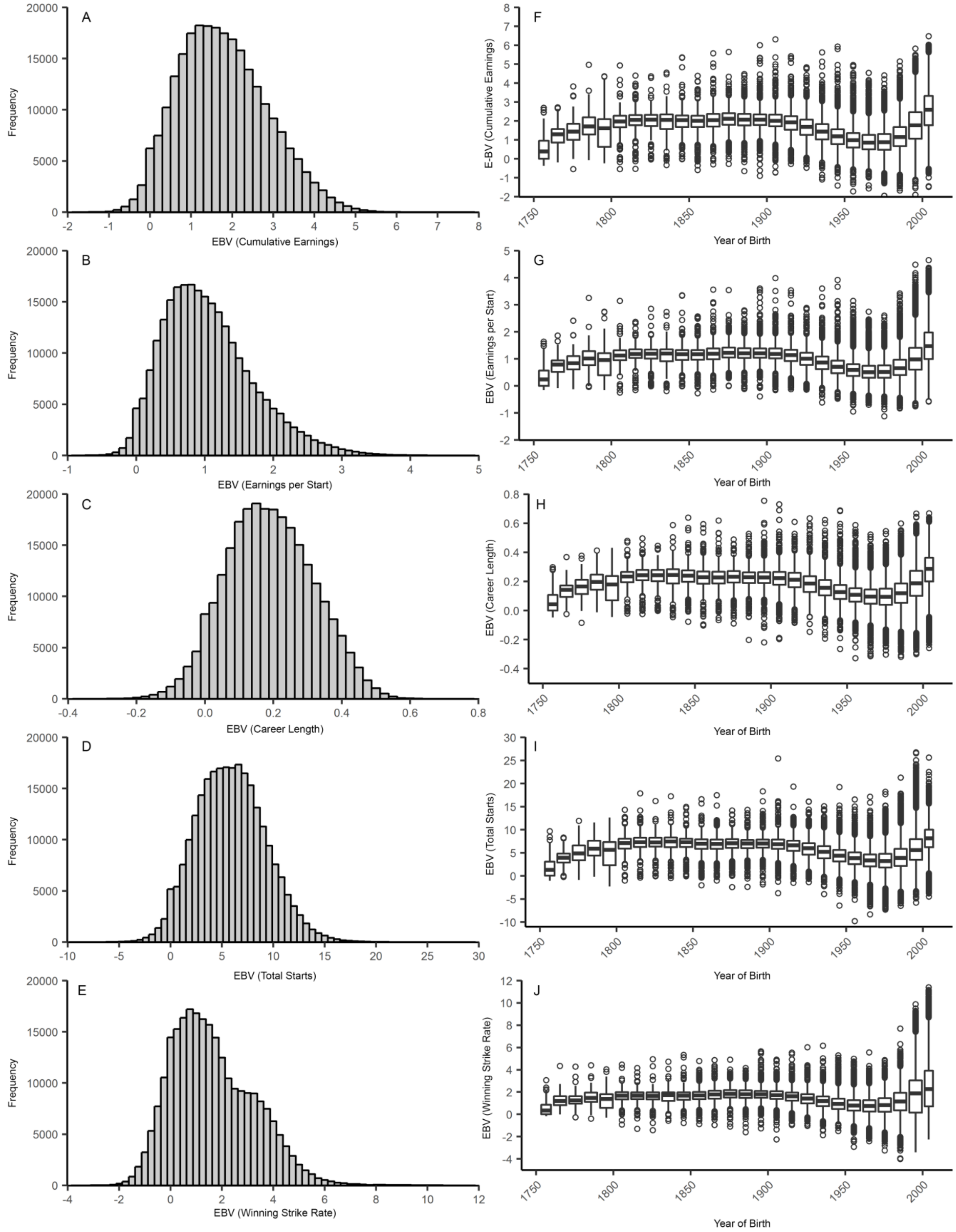

Fig. S6: Selective breeding has increased estimated breeding values (EBVs) in the Australian Thoroughbred population (*n*=257,249). Frequency distributions depict the EBVs for cumulative earnings (A), earnings per start (B), career length (C), total starts (D) and winning strike rate (E) measures. Box and whisker plot of the distributions of EBVs over time are also given for cumulative earnings (F), earnings per start (G), career length (H), total starts (I) and winning strike rate (J).

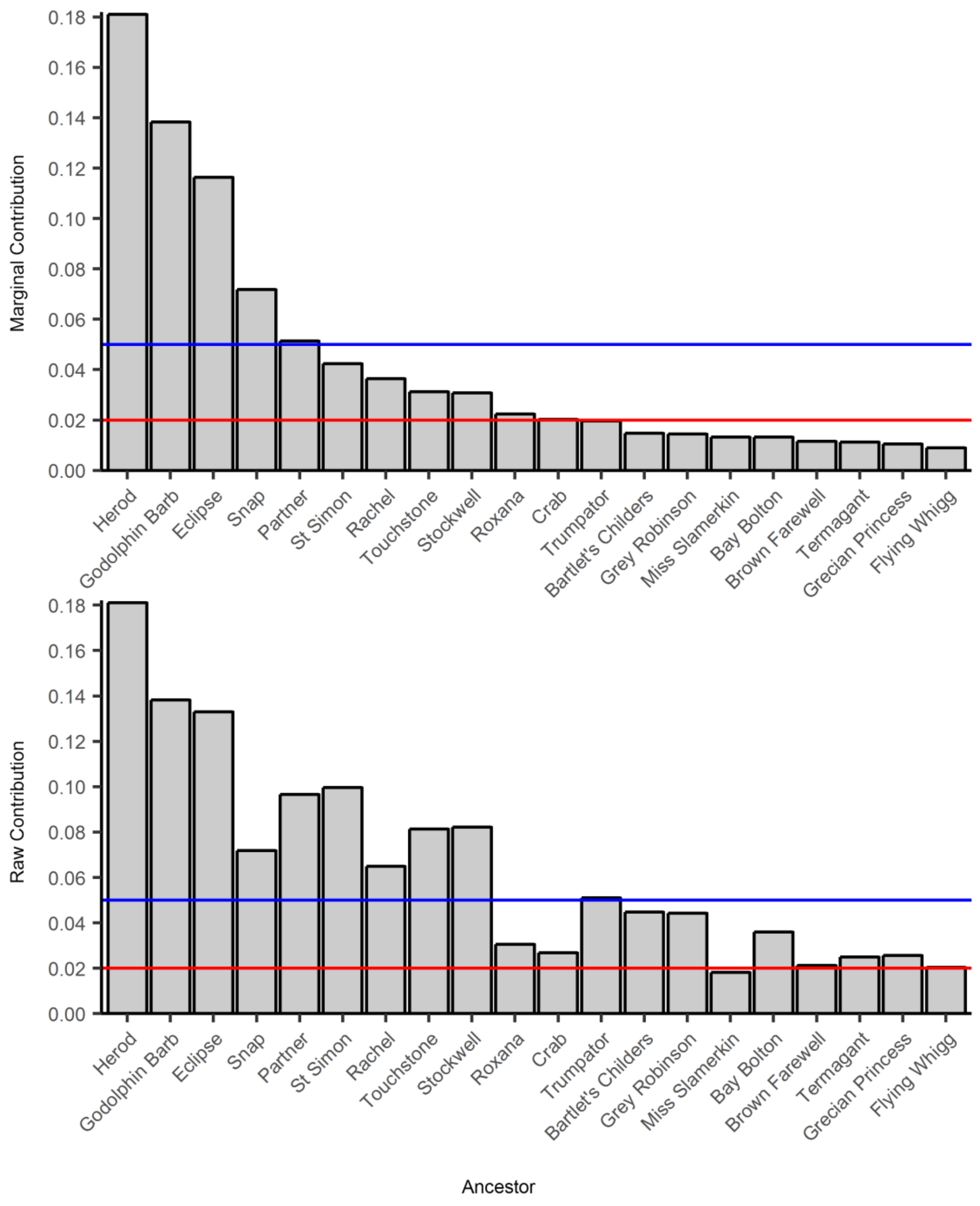

Fig. S7: The top 20 marginal contributors to the contemporary Thoroughbred population (n=135,572). These ancestors were selected using their marginal contributions (A), which can deviate from their raw contribution (B). The blue line indicates the 5% marginal contribution level and the red line represents the 2% level.

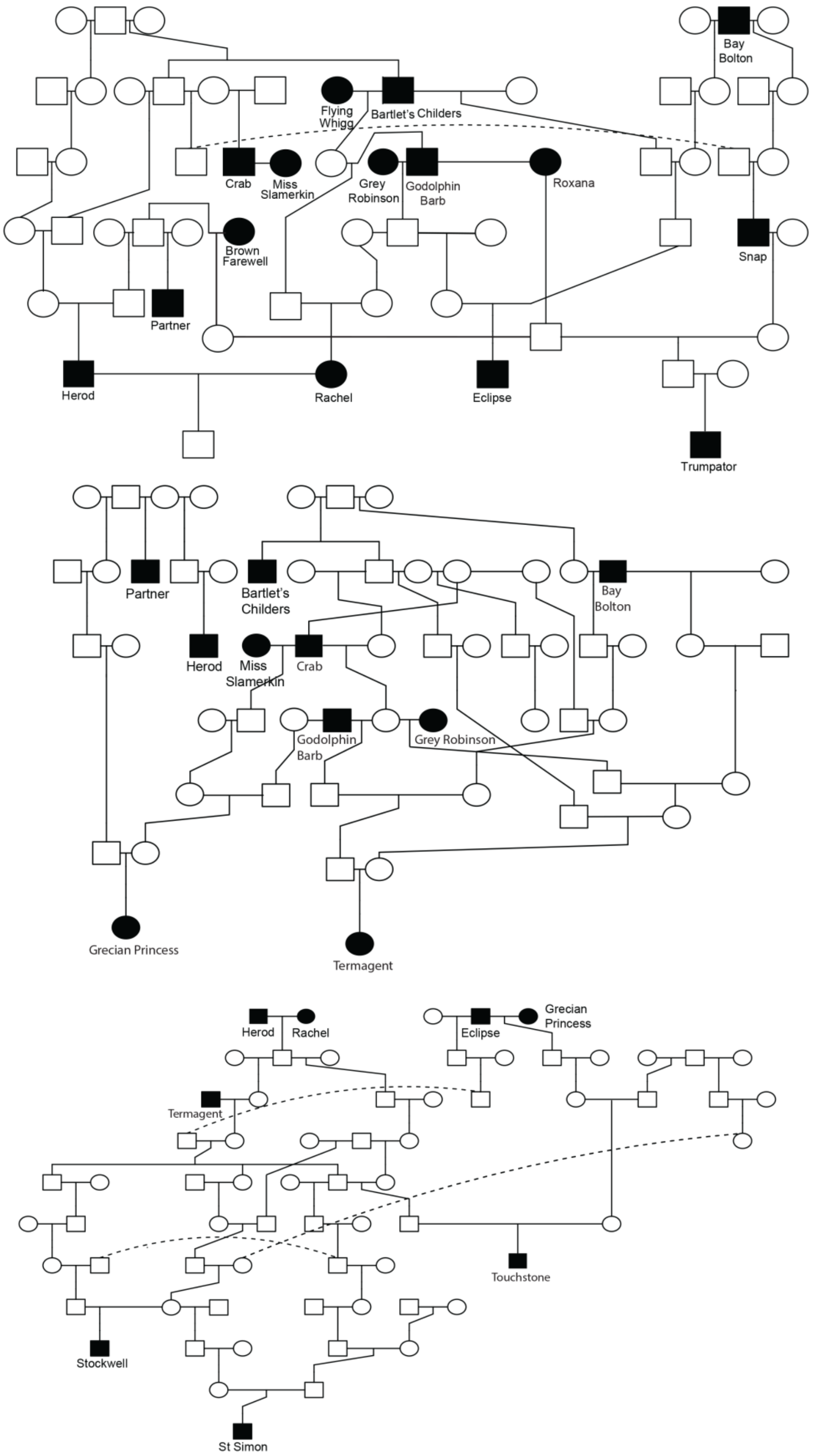

Fig. S8: Pedigrees showing the relationships between the 20 greatest marginal contributors to the Australian Thoroughbred population.

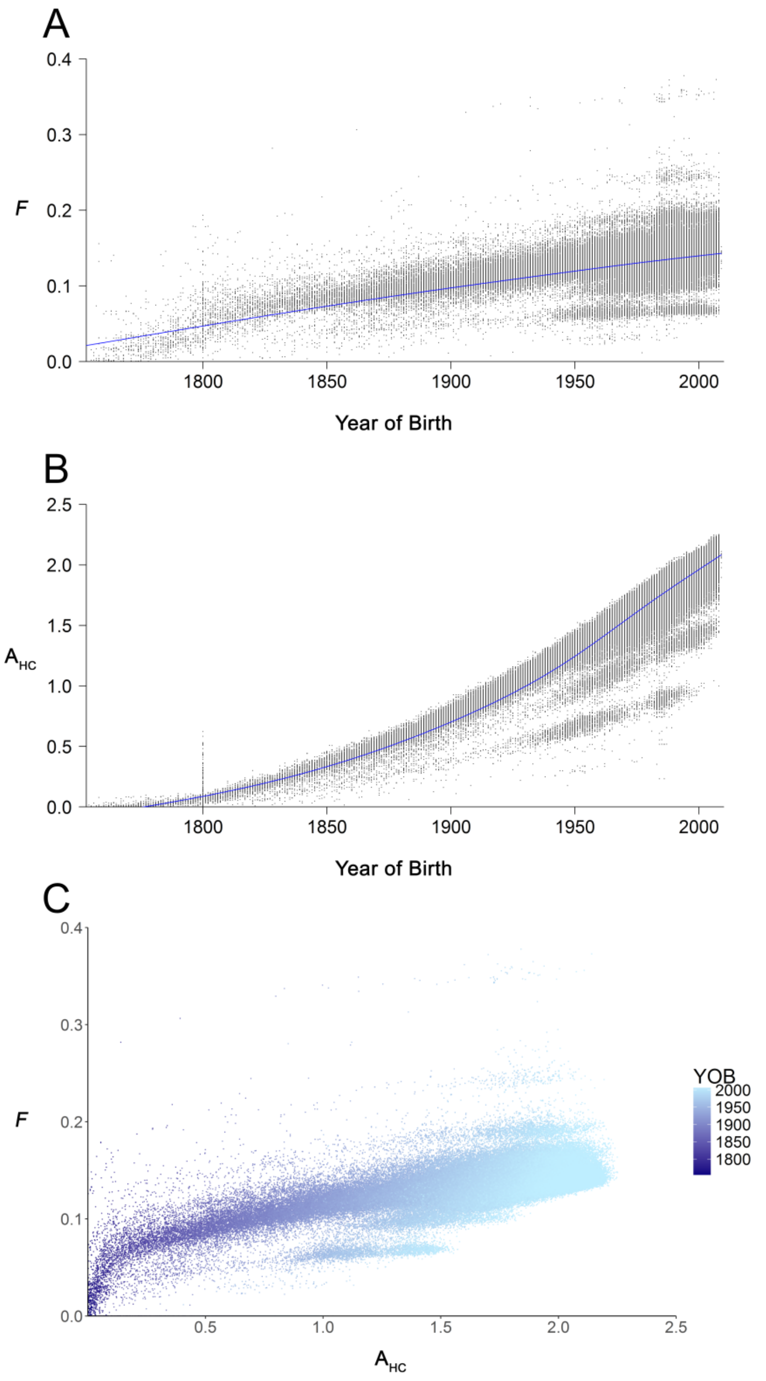

Fig. S9: Inbreeding and purging trends in the Australian Thoroughbred population dating from the foundation of the breed in 1753 to the modern 21st century population (n=500,477). (A) Changes in the levels of F over time in the population. The regression line shows the increasing trend in inbreeding over time. (B) The distribution of A_{HC} levels over time in the population. The regression line shows the accumulation of alleles identical by descent multiple times in the pedigree. (C) F versus A_{HC} with year of birth included as a sliding colour scale.

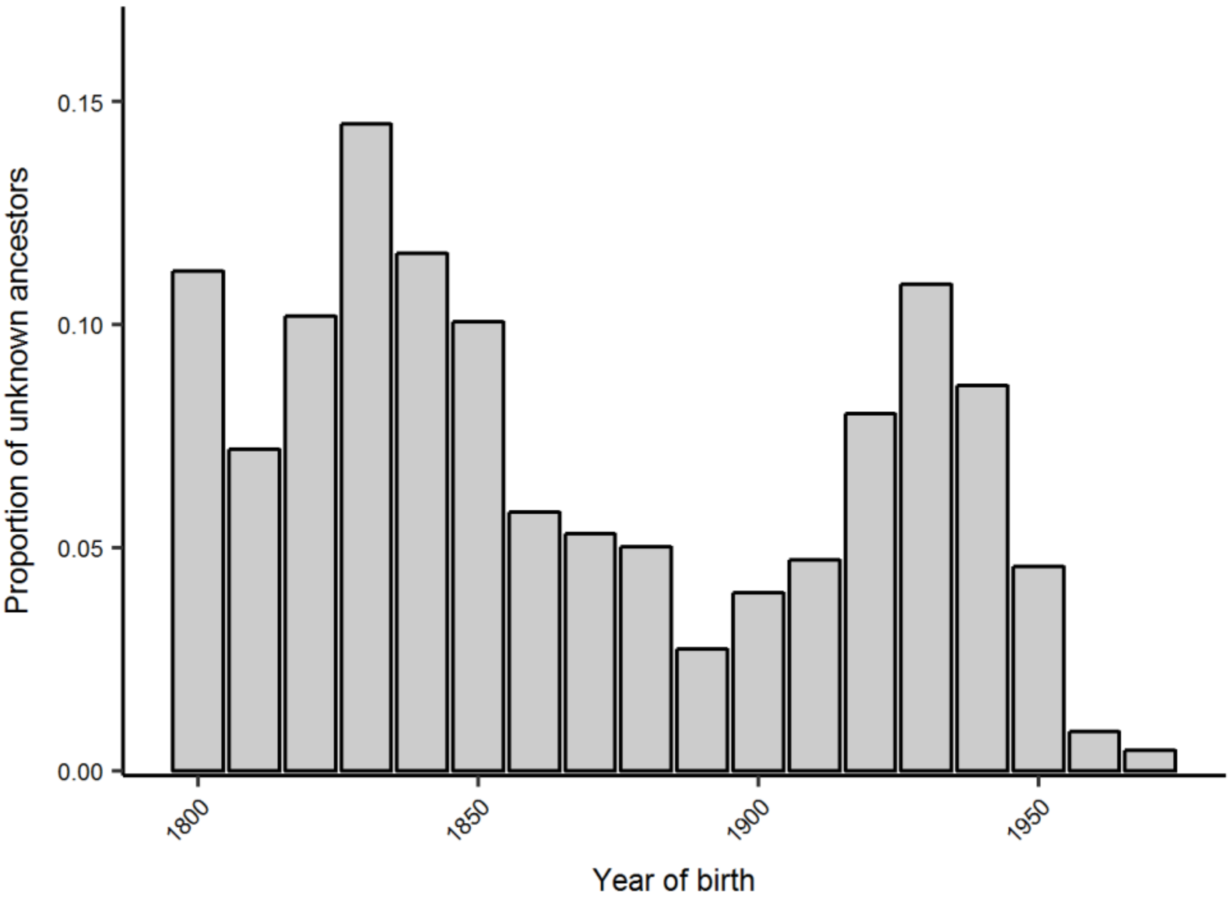

Fig. S10: Proportion of ancestors with incomplete pedigree information from the genealogy of the Australian Thoroughbred horses that were used in the racing performance analysis (*n*=119,637). The date of birth for these individuals was listed as after the studbook was closed (in 1792), so are not considered to be founders of the Thoroughbred population. Data points are given as the proportion of individuals with missing pedigree information from all individuals born over a 10-year period.

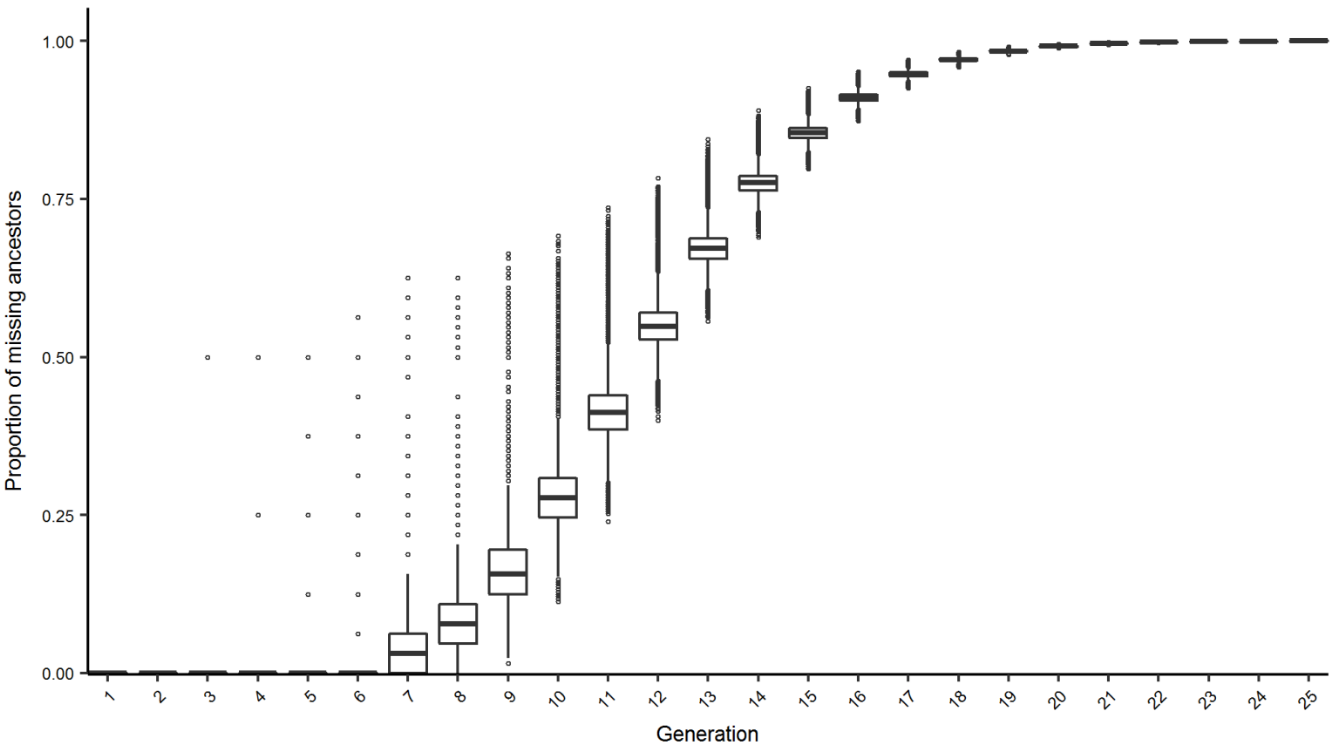

Fig. S11: Proportion of unknown ancestors (*n*=119,637) over 25 generations for all Thoroughbred horses that were used in the analysis of racing performance (*n*=135,572). Proportions were estimated as the number of individuals with pedigree information listed as 0, divided by the total number of individuals found in each generation.

Table S1: The marginal and raw contributions for the top 20 ancestors for the current population (*n*=135,572), selected based on their marginal contributions.

Name	Year of birth	Raw	Marginal
Herod	1758	0.1811	0.1811
Godolphin Barb	1724	0.1383	0.1383
Eclipse	1764	0.133	0.1164
Snap	1718	0.0718	0.0718
Partner	1718	0.0966	0.0514
St Simon	1881	0.0996	0.0423
Rachel	1763	0.065	0.0364
Touchstone	1831	0.0813	0.0313
Stockwell	1849	0.0822	0.0307
Roxana	1718	0.0305	0.0224
Crab	1722	0.0268	0.0202
Trumpator	1782	0.0511	0.0196
Bartlet's Childers	1716	0.0447	0.0147
Grey Robinson	1723	0.0442	0.0145
Miss Slamerkin	1729	0.0182	0.0133
Bay Bolton	1705	0.036	0.0132
Brown Farewell	1753	0.0212	0.0116
Termagant	1772	0.0249	0.0112
Grecian Princess	1770	0.0256	0.0105
Flying Whigg	1715	0.0204	0.009
